# Effectiveness and safety of ranibizumab in patients with central retinal vein occlusion: results from the real-world, global, LUMINOUS study

**DOI:** 10.1038/s41433-021-01702-y

**Published:** 2021-07-29

**Authors:** Andrew Lotery, Andreas Clemens, Raman Tuli, Xun Xu, Masahiko Shimura, Marco Nardi, Focke Ziemssen, Cornelia Dunger-Baldauf, Ramin Tadayoni, on behalf of the LUMINOUS™ study group

**Affiliations:** 1 Clinical and Experimental Sciences, Faculty of Medicine, University of Southampton, Southampton, UK; 2 Novartis Pharma AG, Basel, Switzerland; 3 Department of Cardiology and Angiology I, Heart Center Freiburg University, Faculty of Medicine, University of Freiburg, Freiburg, Germany; 4 Department of Ophthalmology, University of Ottawa, Ottawa, Canada; 5 Department of Ophthalmology, Shanghai First People’s Hospital Affiliated to Shanghai Jiaotong University, Shanghai, China; 6 Department of Ophthalmology, Tokyo Medical University Hachioji Medical Center, Hachioji-shi, Tokyo-To Japan; 7 Ophthalmology Unit, University of Pisa, Pisa, Italy; 8 Department for Ophthalmology, Eberhard Karl University Tübingen, Tübingen, Germany; 9 Université de Paris, Ophthalmology Department, AP-HP, Lariboisière, Saint Louis and Fondation Rothschild Hospitals, Paris, France

**Keywords:** Retinal diseases, Clinical pharmacology

## Abstract

**Objective:**

To evaluate the effectiveness, treatment patterns and long-term safety of ranibizumab 0.5 mg in treatment-naïve patients with central retinal vein occlusion (CRVO) in a real-world setting.

**Methods:**

LUMINOUS, a 5-year, global, prospective, multicentre, multi-indication, observational, open-label study, recruited treatment naïve or prior treated patients who were treated as per the local ranibizumab label. Here, we report the mean change in visual acuity (VA; Early Treatment Diabetic Retinopathy Study [ETDRS] letters), treatment exposure over year (Y) 1 and 5-year safety in treatment-naïve CRVO patients.

**Results:**

At baseline, the mean age of treatment-naïve CRVO patients (*n* = 327) was 68.9 years, with a mean (Standard deviation [SD]) VA of 40.6 (23.9) letters. At Y1, patients (*n* = 144) had a mean (SD) VA gain from baseline of 10.8 (19.66) letters, with a mean (SD) of 5.4 (2.65) ranibizumab injections. Patients demonstrated mean (SD) VA gains of 2.7 (19.35), 11.6 (20.56), 13.9 (18.08), 11.1 (18.46) and 8.2 (24.86) letters with 1, 2–3, 4–5, 6–8 and >8 ranibizumab injections, respectively. Mean (SD) VA gains at Y1 in patients receiving loading (67.4%) and no loading dose (32.6%) was 11.9 (20.42) and 8.4 (17.99) letters, respectively. Over five years, the incidence of ocular/non-ocular adverse events (AEs) and serious AEs was 11.3%/8.6% and 1.2%/6.7%, respectively.

**Conclusions:**

These results demonstrate the effectiveness of ranibizumab in treatment-naïve CRVO patients at Y1 with clinically meaningful VA gains and no new safety findings over five years. These findings may help inform routine practice and enable better clinical management to achieve optimal visual outcomes.

## Introduction

The global prevalence of central retinal vein occlusion (CRVO) is 0.08% in patients aged ≥ 30 years and does not significantly vary with regard to race or gender [1–3]. Anti-vascular endothelial growth factor (anti-VEGF) therapy is the current standard of care for the treatment of CRVO patients [4–8].

Ranibizumab (Lucentis^®^; Novartis Pharma AG, Basel, Switzerland, and Genentech Inc., South San Francisco, CA, USA) was the first anti-VEGF agent to be approved for the treatment of patients with visual impairment due to macular oedema secondary to retinal vein occlusion (branch and central) and is currently approved in many countries globally for this indication [9–11]. The efficacy and safety profile of ranibizumab in patients with CRVO is well established based on several randomised controlled trials [9, 10, 12]. However, real-world evidence is limited to specific regions, countries or small patient populations [13–17].

Hence, the LUMINOUS (NCT01318941) study is, to our knowledge, the largest, prospective, observational, global trial in the field of medical retina designed to evaluate the long-term effectiveness, safety and treatment patterns associated with ranibizumab 0.5 mg in routine clinical practice across five approved indications: (i) neovascular age-related macular degeneration, (ii) diabetic macular oedema, (iii) branch retinal vein occlusion, (iv) CRVO and (v) myopic choroidal neovascularisation. The effectiveness of ranibizumab at one year and safety over five years for treatment-naïve patients with CRVO enroled in this study are reported here.

## Materials and methods

### Study design

LUMINOUS was a 5-year, open-label, single-arm, global, observational study conducted from March 2011 to April 2016 at 488 clinical sites across 42 countries. Patients with any of the approved indications as per the ranibizumab label at the time of the study were enroled and treated at outpatient or private ophthalmology clinics with intravitreal ranibizumab 0.5 mg according to the local ranibizumab label [18]. As patients were recruited over time and the date of study completion was pre-set, the follow-up time varied between patients based on their study entry date. The minimum potential follow-up for each patient was defined as one year in the protocol. The frequency of patient visits was determined by the investigator. It was recommended to capture data at every visit or at a minimum of every three months. Investigators were encouraged to follow-up with patients who did not visit the clinic for at least six months since their last visit. Patients who were not seen at least once per year or those who switched to another anti-VEGF therapy were discontinued from the study.

The study protocol was reviewed and approved by an Independent Ethics Committee or Institutional Review Board for each centre; a complete list of these by study centre is provided in the Supplementary Table 1. The study was conducted in accordance with the Guidelines for Good Pharmacoepidemiology Practices issued by the International Society for Pharmacoepidemiology [19] and any applicable national guidelines and ethical principles laid down in the Declaration of Helsinki. Patients provided written informed consent. The study is registered with ClinicalTrials.gov as NCT01318941 [20].

### Key eligibility criteria

The key inclusion criteria for the LUMINOUS study have been described previously [21–24]. Patients were excluded if they were simultaneously participating in a study that included administration of any investigational drug or procedure, had undergone systemic treatment with any VEGF inhibitor in the 90 days prior to study enrolment or had received ocular treatment with any anti-VEGF other than ranibizumab in the month prior to study enrolment.

### Assessments

Effectiveness assessments included visual acuity (preferably best-corrected visual acuity [VA]) evaluation by each participating physician as a part of routine care practice using Early Treatment Diabetic Retinopathy Study (ETDRS) letters, Snellen charts or equivalent. To facilitate data analysis, Snellen fractions and decimals were converted to the ETDRS equivalent letter scores. It was recommended that the same method of visual acuity assessment be used throughout the study wherever possible.

Other assessments, such as optical coherence tomography (i.e. for central retinal thickness evaluation) and ocular examinations (pre-injection intraocular pressure measurements), were optional but included if the data were available. The number of ranibizumab injections administered overall and over time; the average time interval (in weeks) between consecutive injections; visit frequency; and treatment patterns (unilateral [involving single eye]/bilateral [involving both eyes]) were recorded. All adverse events (AEs), including serious AEs (SAEs), that occurred during the study were recorded irrespective of suspected causal association.

### Statistical analysis

All effectiveness and safety data were summarised descriptively. Owing to the design of the study, one year data were potentially available for all patients, while the availability of data for subsequent years depended on the patient’s study entry date. The effectiveness data are therefore presented here for up to one year. Safety data are presented over the entire 5-year period to provide a comprehensive safety profile.

The enroled set included all patients who signed the informed consent and had at least a baseline assessment. The safety set comprised patients in the enroled set, who, in case of treatment naïve patients, were treated with at least one dose of ranibizumab during the study and had at least one safety assessment after the first injection.

The primary treated eye set included all primary treated eyes (i.e. the first eye treated during the study) in patients from the safety set. Treatment exposure up to one year was analysed for patients in the safety set who stayed in the study for at least 365 days. For year 1 effectiveness, patients from the primary treated eye set who had both baseline and year 1 data and remained in the study for at least 365 days are presented.

The primary effectiveness end point was the mean change in VA ETDRS letter score from baseline to year 1. Additional effectiveness analyses not prespecified in the protocol but included in the statistical analysis plan are: (1) the mean change in VA from baseline at year 1 by (a) injection frequency during year 1 (1, 2–3, 4–5, 6–8 and >8 injections); (b) patients who received a loading dose (the initial three ranibizumab injections administered up to day 100) versus those who did not; (c) baseline VA category (<23, 23 to <39, 39 to <60, 60 to <74 and ≥74 letters); (d) baseline VA < 73 letters or ≥73 letters (good starting vision or Snellen equivalent 20/40) at year 1; (2) proportion of patients with VA loss (defined as ≤0-letter change from baseline) or gain (defined as >0-letter change from baseline) of >0 to <5 letters, 5 to <10 letters, 10 to <15 letters and ≥15 letters at year 1.

Safety was assessed based on the incidence, proportion, relationship and severity of treatment-emergent ocular and non-ocular AEs. Ocular AEs were assessed for the primary treated eye set, and non-ocular AEs were assessed for the safety set.

## Results

A total of 30,138 patients were enroled across all five approved indications in the overall LUMINOUS trial. Of the total population, 1048 (3.5%) patients had CRVO, of whom 327 (31.2%) were treatment naïve. The following countries enroled the majority of treatment-naïve patients with CRVO: the United Kingdom (24.5%), Canada (13.1%), Russia (11.0%), Germany (9.5%) and Poland (9.4%).

**Fig. 1:**
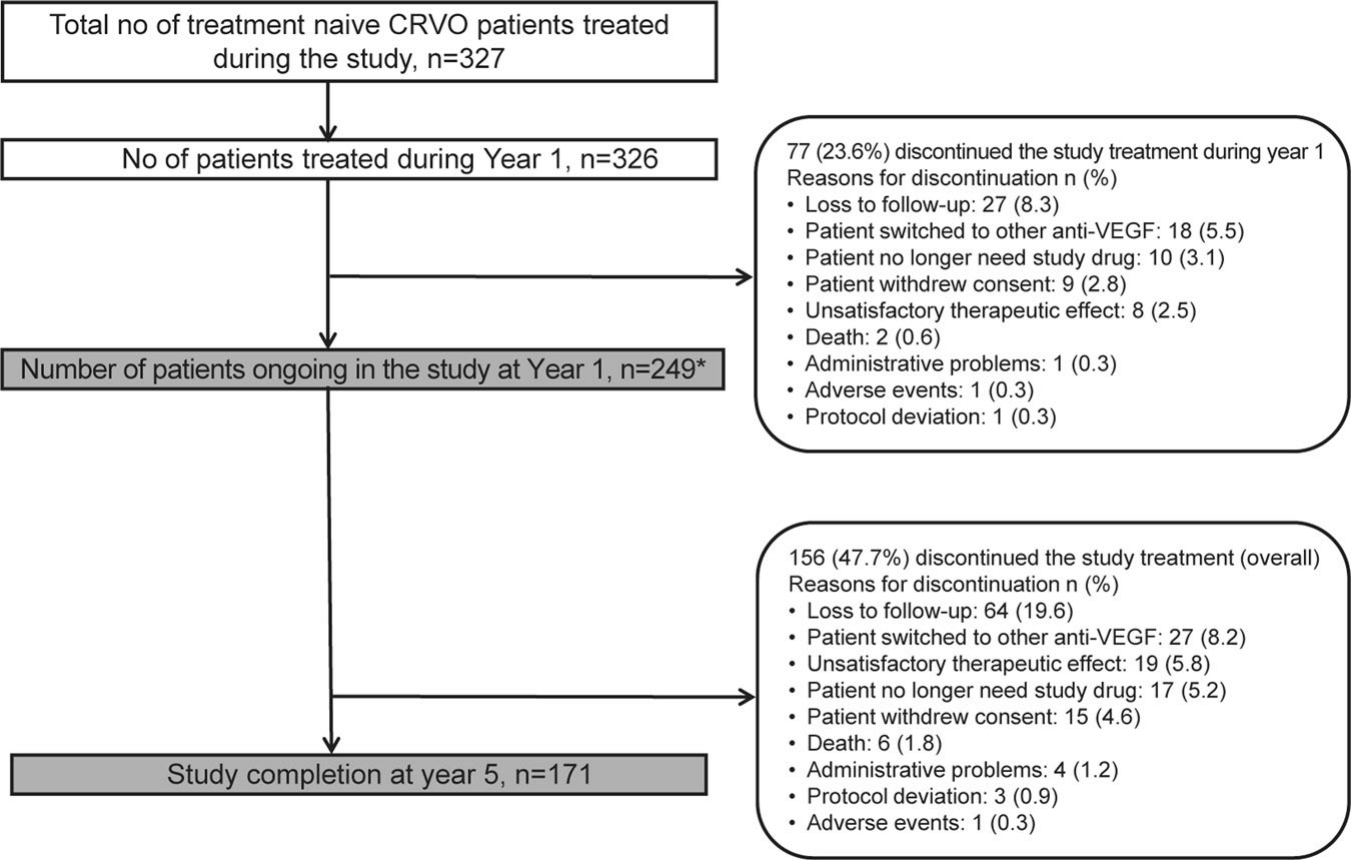
Patient disposition. Safety set. *Number of patients continuing in the study at year 1. Year 1 discontinuation rates are also included in the overall discontinuation rate. CRVO central retinal vein occlusion, n number of patients, VEGF vascular endothelial growth factor.

Of the 327 treatment-naïve patients, 249 (76.14%) remained in the study until the end of year 1, and 171 (52.3%) completed the study. The most common reason for study discontinuation was ‘loss to follow-up’ at year 1 (*n* = 27, 8.3%) and year 5 (*n* = 64, 19.6%; Fig. 1). The year 1 effectiveness analysis in the primary treated eye set included 144 patients for whom both baseline and 1-year VA data were available (owing to the flexible scheduling of visits, not all patients who completed year 1 in the study had a year 1 visit).

At baseline, the mean (standard deviation [SD]) age of the patients was 68.9 (13.0) years, 56.9% were male, and the majority (76.8%) were White. Most patients (*n* = 323, 98.78%) were treated in one eye, and four (1.22%) patients were treated bilaterally. No eminent differences in baseline characteristics were noted when comparing the overall treatment-naïve CRVO population, patients included in the year 1 effectiveness analysis and those not included due to unavailable data or participation in the study for <365 days (Table 1).

**Table 1:** Baseline demographic and ocular characteristics for treatment-naïve patients with CRVO.

Characteristics	Overall	Patients with Year 1 data	Patients without Year 1 data
	*N* = 327^a^	*n* = 144^b^	*n* = 183^c^
Mean (SD) age, years	68.9 (13.0)	70.0 (11.88)	67.9 (13.83)
Gender, (%)			
Male	186 (56.9)	74 (51.4)	112 (61.2)
Race, (%)			
White	251 (76.8)	122 (84.7)	129 (70.5)
Asian	38 (11.6)	9 (6.3)	29 (15.8)
Native American	2 (0.6)	0	2 (1.1)
Black	1 (0.3)	0	1 (0.5)
Other	19 (5.8)	3 (2.1)	16 (8.7)
Missing	16 (4.9)	10 (6.9)	6 (3.3)
VA			
*n*	294	144	150
Mean (SD) VA, ETDRS letters	40.6 (23.9)	40.7 (22.17)	40.6 (25.44)
CRT			
*n*	224	109	115
Mean (SD) CRT, µm	551.5 (220)	566.4 (206.29)	537.4 (232.17)
Median time from diagnosis to first treatment, days	21	16.5	23

*CRT* central retinal thickness, *CRVO* central retinal vein occlusion, *EDTRS* early treatment diabetic retinopathy study, *n* number of patients, *SD* standard deviation, *VA* visual acuity

^a^All patients who signed the informed consent and had at least a baseline assessment (enroled set).

^b^Patients included in Year 1 effectiveness analysis who had both baseline and Year 1 data and had remained in the study for at least 365 days (primary treated eye set).

^c^Patients who have unavailable baseline or Year 1 VA data, or are not included in effectiveness analysis for Year 1 or have been in the study for <365 days (primary treated eye set).

At baseline, the mean age, mean VA and median time from diagnosis to treatment varied across the top five recruiting countries (Supplementary Fig. 1). The median time from diagnosis to first treatment was one day in Canada and Poland, eight days in Germany, 30 days in the United Kingdom and 48.5 days in Russia.

### Treatment exposure and visits

**Fig. 2:**
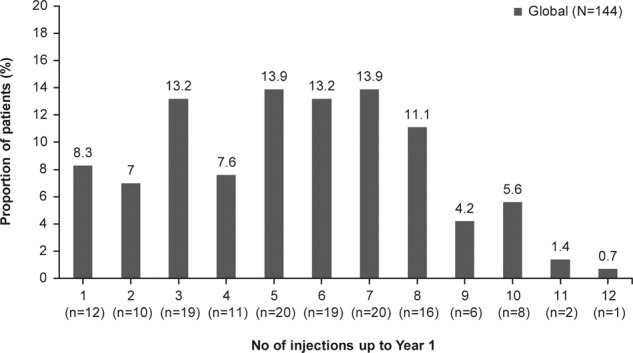
Frequency of ranibizumab injections over 1 year (year 1 primary treated eye set). The year 1 primary treated eye set included patients from the primary treated eye set who had both baseline and year 1 data and remained in the study for at least 365 days.

The mean (SD) number of ranibizumab injections administered up to year 1 was 5.4 (2.65), and the mean (SD) number of visits was 8.7 (2.98). In the first year, 50.1% of patients received six or more injections (Fig. 2).

### Effectiveness outcomes

**Fig. 3:**
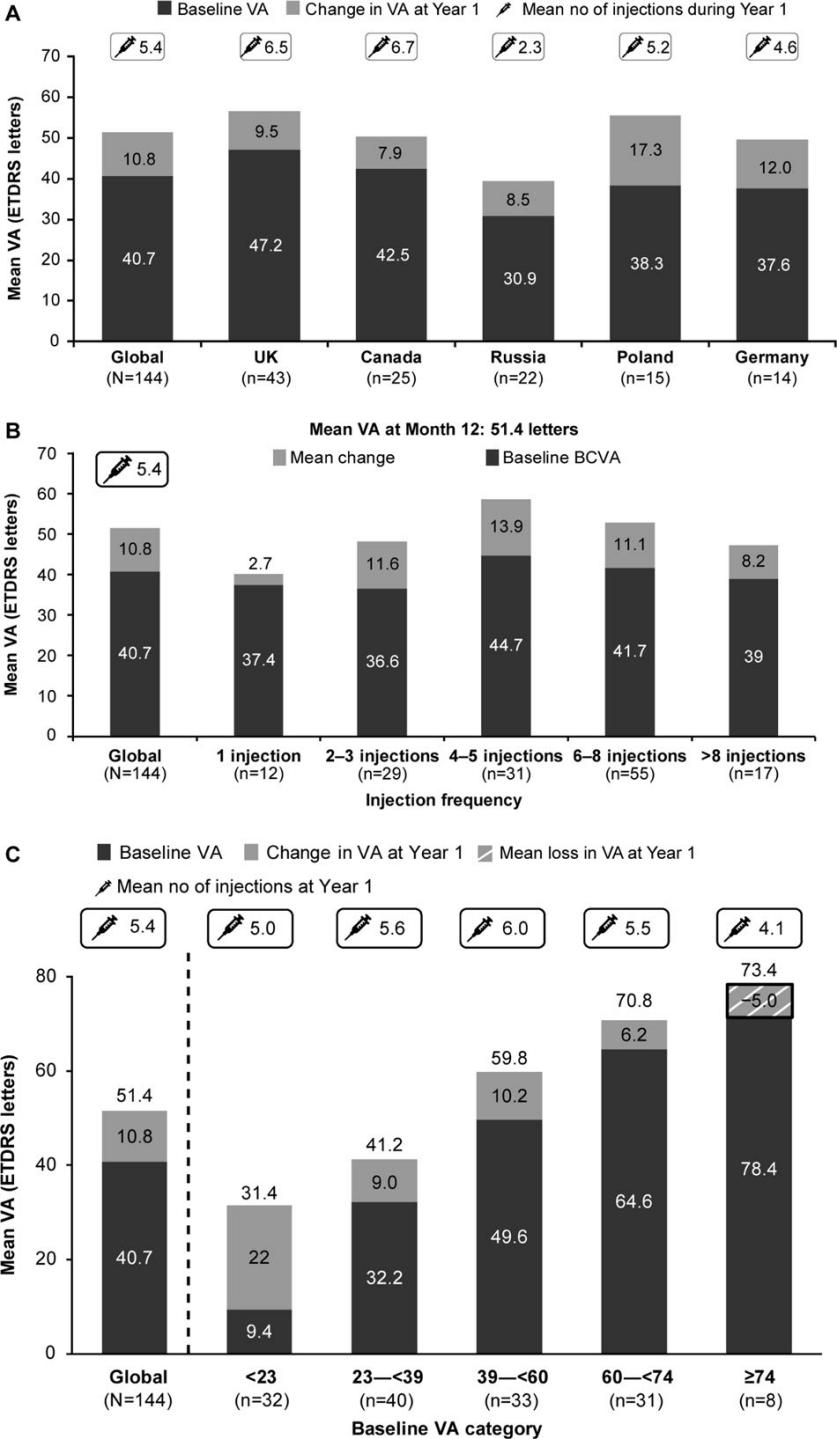
Mean change in VA (letters) from baseline to year 1 (year 1 primary treated eye set). **A** For global and the top five enroling countries. **B** By injection frequency. **C** By baseline VA category. The year 1 primary treated eye set included patients from the primary treated eye set who had both baseline and year 1 data and had remained in the study for at least 365 days. ETDRS early treatment diabetic retinopathy study, n number of patients, VA visual acuity.

At year 1, a mean (SD) VA gain of 10.8 (19.7) letters from a baseline of 40.7 (22.17) letters was observed (Fig. 3A). The mean VA gain and the mean number of ranibizumab injections at year 1 varied across the enroling countries (Fig. 3A).

By injection category, the highest mean VA gains from baseline to year 1 were observed in patients receiving 4–5 injections. Across different injection categories, the mean (SD) VA gains at year 1 ranged from 2.7 (19.35) to 13.9 (18.08) (Fig. 3B). By baseline VA category, patients with a lower VA (<23 letters) at baseline had higher VA gains at year 1 (*n* = 32; baseline VA: 9.4 [8.35]; VA gain at year 1: 22.0 [23.02] letters); however, the actual VA at year 1 was higher in patients with a higher baseline VA. Eight patients (baseline VA: 78.4 [4.41] letters) with baseline VA ≥ 74 letters showed a mean (SD) change in their vision at year 1 by −5.0 (7.52) letters. The mean number of injections by the end of year 1 ranged from 4.1 to 6.0 across different baseline VA categories (Fig. 3C).

VA gains in patients who received the loading dose of three initial consecutive monthly ranibizumab injections (*n* = 97 of 144; 67.4%) was [11.9 (20.42)] letters and in those who did not was 8.4 (17.99) letters; 95% CI (confidence interval) for the difference (─3.14, 10.14) (Supplementary Fig. 2).

At month 6 and year 1, 46.5% (*n* = 60, *N* = 129) and 44.4% (*n* = 64, *N* = 144) of patients treated with ranibizumab had VA gains of ≥15 letters, respectively. At year 1, VA was maintained at baseline levels in 7.6% (*n* = 11) of patients; a VA loss of ≥15 letters was seen in 11.8% (*n* = 17) of patients (Supplementary Fig. 3).

### Safety outcomes

At the end of year 1, the incidence of ocular/non-ocular AEs in the 144 patients analysed was 8.3% (*n* = 12)/5.6% (*n* = 8), and that of the SAEs was 0.7% (*n* = 1)/4.2% (*n* = 6). Over the 5-year period, ocular AEs in the safety set (*n* = 327) were reported in 11.3% (*n* = 37) of patients. Glaucoma (*n* = 5, 1.5%), ocular hypertension (*n* = 4, 1.2%) and cataract (*n* = 4, 1.2%) were the most commonly reported ocular AEs (Table 2). Non-ocular AEs were reported in 8.6% (*n* = 28) of patients; hypertension, pneumonia and confusional state were the most common, occurring in two patients each (*n* = 2, 0.6%). Overall, 3.4% (*n* = 11) of ocular AEs were suspected to be related to the study treatment or ocular injections by the investigator (Supplementary Table 2). Non-ocular treatment-related AEs suspected to be related to treatment were not observed. One ocular AE (0.3%, retinal injury) and six non-ocular AEs (all SAEs) led to treatment discontinuation.

**Table 2:** Incidence of ocular and non-ocular AEs over 5 years (safety set).

Preferred term, *n* (%)	Treatment-naïve patients with CRVO
	*N* = 327
Ocular AEs, total	37 (11.3)
Glaucoma	5 (1.5)
Ocular hypertension	4 (1.2)
Cataract	4 (1.2)
Eye pain	3 (0.9)
Conjunctival haemorrhage	3 (0.9)
IOP increased	2 (0.6)
Conjunctivitis	2 (0.6)
Visual acuity reduced	2 (0.6)
Retinal vein occlusion	2 (0.6)
Vitreous detachment	2 (0.6)
Non-ocular AEs, total	28 (8.6)
Confusional state	2 (0.6)
Pneumonia	2 (0.6)
Hypertension	2 (0.6)

Ocular and non-ocular AEs ≥2 patients are shown. Preferred terms are presented by descending order of frequency. A patient with multiple occurrences of an AE was counted once. A patient with multiple AEs was also counted only once. Patients with a baseline visit date present are included. Data collected until the last recorded follow-up date was used to perform the analyses.

*AE* adverse events, *CRVO* central retinal vein occlusion, *N* total number of patients, *n* number of patients.

Ocular SAEs were reported in four (1.2%) patients: glaucoma, *n* = 2 (0.6%) and amaurosis fugax, blindness and cataract trauma, *n* = 1 each (0.3%) (Supplementary Table 3). No cases of endophthalmitis or retinal break/detachment were reported. The incidence of non-ocular SAEs was 6.7% (*n* = 22); pneumonia and confusional state (0.6%; *n* = 2 each) were the most frequently reported non-ocular SAEs (Supplementary Table 3). In total, there were six deaths, with reasons including breast cancer, pneumonia, sepsis and unknown cause.

## Discussion

The results from the global, prospective LUMINOUS study support the safety of ranibizumab 0.5 mg treatment over 5 years in treatment-naïve patients with CRVO in a broad real-world setting. Ranibizumab treatment resulted in improved VA outcomes at the end of 1 year in these patients. Across all enroling countries we identified a similar pattern, i.e. that patients with a low baseline VA or those receiving higher numbers of ranibizumab injections or those treated with an adequate loading dose achieved better VA outcomes. Overall, there were no new safety findings, and very few AEs, SAEs or AEs leading to treatment discontinuation and treatment-related AEs were reported. The safety results observed in the LUMINOUS study are consistent with those observed in other CRVO studies with ranibizumab [10, 11, 25, 26]. These findings may help inform routine practice and enable better clinical management to achieve optimal visual outcomes in treatment-naïve CRVO patients. Although efficacy could not be assessed on the basis of anatomical response, the observed influences of consistent upload/start of therapy highlight the risk of limited visual acuity gain under hesitant therapy as also found by Sagkriotis et al. [27].

The mean age (years) of patients in this study (68.9) was similar to that of patients included in the CRUISE (67.6) [10, 11], CRYSTAL (65.5) [9] and OCEAN (70.3) [28, 29] studies. Furthermore, the majority of treatment-naïve patients with CRVO included in LUMINOUS were White (77%), consistent with other CRVO studies with ranibizumab (83.1–94.4%) [9–11, 28]. Patients in the LUMINOUS study had a lower baseline VA (40.7 letters), similar to the CRVO treatment arm of the OCEAN study (43.7 letters) [28, 29], when compared with the CRUISE (48.3 letters) and CRYSTAL (53.0 letters) studies. A lower baseline VA is expected to result in higher VA gains. However, the overall VA gains were lower in LUMINOUS (10.8 letters) than in the CRYSTAL (12.3 letters) and CRUISE (13.9 letters) studies. These lower VA gains were in line with the real-world OCEAN study [29]. While this difference could possibly be due to the lower mean number of injections in the LUMINOUS (5.4) and OCEAN (5.11) [29] studies compared with the CRUISE (8.9) [10, 11] and CRYSTAL (8.1) [9] studies, between-trial comparisons have significant limitations when considering the differences in their study designs.

Gains in VA with treatment were dependent on the patient’s baseline VA as well as on the early initiation of treatment after diagnosis of the disease in studies [10, 16, 30–38]. In the LUMINOUS study, results by baseline VA categories support the observations made in phase 3 studies of ranibizumab in patients with CRVO (CRUISE and CRYSTAL) [9–11], suggesting that patients with a poor baseline VA achieve higher VA gains. However, the actual VA at year 1 in the LUMINOUS study as well as in the phase 3 studies, was higher in patients with a better baseline VA, stressing the need for early diagnosis and prompt treatment of the disease.

Very few real-world studies have investigated ranibizumab in treatment-naïve patients with CRVO. In a retrospective, observational, multicentre study carried out in Portugal, 76 treatment-naïve patients with CRVO were included and treated with ranibizumab or bevacizumab. The median age of patients and distribution of gender were comparable to those in the LUMINOUS study, but the baseline VA and VA at month 12 in patients treated with ranibizumab were higher compared with those in the LUMINOUS study (median VA at baseline: 0.7 logarithm of the minimum angle of resolution [~65 ETDRS letters] and median VA at 12 months: 0.5 logarithm of the minimum angle of resolution [~75 letters]); the median number of injections up to month 12 was four. Consistent with the findings in LUMINOUS, patients with a lower baseline VA had better gains at 12 months [16].

The strengths of the LUMINOUS study are that it is the first large-scale, global, multicentre, multi-indication, post-market, observational study of an anti-VEGF agent, with flexible inclusion criteria. LUMINOUS included a broader patient population than other randomised controlled trials and provides information on patients with multiple geographic, demographic and baseline characteristics, with varying degrees of healthcare access. It allowed evaluating the hypotheses of positive or negative outcomes in the different care systems (i.e. time to first treatment, application of the complete loading dose, etc.). Limitations include a potential selection bias by analysing patients for effectiveness who were ongoing in the study at one year and had a one-year visit. The restriction was implemented to be in a position to compare these results with other studies which showed one-year results. It is difficult to assess the direction of the bias. Information bias was expected to be minimal owing to systematic site training, the use of standardised case report forms and other guidance documentation to ensure consistent data collection. Along with manual data reviews, programmable data edit checks for missing, illogical or out-of-range values were built into the electronic data capture system to ensure data quality at all sites. Potential bias was taken into account for the interpretation of study results. Other limitations include variable treatment schedules across regions, host country healthcare systems, physician’s discretion of inclusion, access to treatment, cost and reimbursement criteria, all of which can lead to under-treatment, suboptimal therapeutic effectiveness and poor patient follow-up potentially affecting outcomes. In addition, the imaging data were not collected uniformly and analysed. The study also lacked a comparator arm; options for comparator arms were very limited at the start of the study. Other intravitreal VEGF-inhibitors were not yet available, and design options for comparing the treatment patterns of ranibizumab 0.5 mg were not in scope as the newly emerging treatment regimens like ‘treat-and-extend’ were not very widespread at the time of the study.

To conclude, the results from the LUMINOUS study support the robust real-world effectiveness of ranibizumab over a 1-year treatment period and safety over a 5-year period in treatment-naïve patients with CRVO. There were no new safety signals identified, and the safety profile was consistent with the known safety profile of ranibizumab.

### Summary

#### What was known before

- Ranibizumab, the first anti-VEGF agent approved for the treatment of patients with visual impairment due to macular oedema secondary to retinal vein occlusion (branch and central) is currently approved in many countries globally for this indication.
- Though the efficacy and safety profile of anti-VEGF treatment in patients with CRVO is well established based on several randomised controlled trials, real-world evidence is limited to specific regions, countries or small patient populations.

#### What this study adds

- LUMINOUS is the largest, prospective, observational study in medical retina and evaluated the long-term effectiveness, treatment patterns, and safety of ranibizumab 0.5 mg across all approved indications in a real-world scenario over 5 years.
- The present article reports the one year effectiveness and five years safety of ranibizumab for treatment-naive patients with CRVO.

## Supplementary information

Supplementary Information
