# Supplementary material for: Effectiveness and safety of ranibizumab in patients with central retinal vein occlusion: results from the real-world, global, LUMINOUS study
Source: Eye (Lond). 2021 Jul 29;36(8):1656–61. doi: 10.1038/s41433-021-01702-y (PMC9307792; doi:10.1038/s41433-021-01702-y)
Supplement: Supplementary file 1 — Supplementary Information [file 41433_2021_1702_MOESM1_ESM.docx]

**Supplementary Table 1. List of Independent Ethics Committees (IEC) or Institutional Review Boards (IRBs) by study center.**

| **Center Number** | **Ethics Committee or Institutional Review Board** | **Department / Organization** | **EC/IRB**  **City** | **EC/IRB**  **State/ Province** | **EC/IRB**  **Postal Code** | **Center Country** |
| --- | --- | --- | --- | --- | --- | --- |
| 1100 | Comite Independiente de Etica para Ensayos en Famacologia Clinica | Consultorio de Investigaciones Oftalmológicas | Buenos Aires | Buenos Aires | C1027AAP | Argentina |
| 1101 | Comité de Ética en Investigación Instituto de Investigaciones Clínicas | Clínica Privada de Ojos José León Suárez | Buenos Aires | Buenos Aires | C1027AAP | Argentina |
| 1102 | Comite Independiente de Etica para Ensayos en Famacologia Clinica | Consultorio Dr. Andres Jakofsky | Buenos Aires | Buenos Aires | C1027AAP | Argentina |
| 1103 | Comite Independiente de Etica para Ensayos en Famacologia Clinica | Clínica de Ojos | Buenos Aires | Entre Rios | C1027AAP | Argentina |
| 1104 | Comite Independiente de Etica para Ensayos en Famacologia Clinica | DYTER S.A. | Buenos Aires | Mendoza | C1027AAP | Argentina |
| 1105 | Comite Independiente de Etica para Ensayos en Famacologia Clinica | Clínica de Ojos Srl | Buenos Aires | Santa Fe | C1027AAP | Argentina |
| 1106 | Comite Independiente de Etica para Ensayos en Famacologia Clinica | Microcirugia Ocular (Clínica MICRO) | Buenos Aires | Santa Fe | C1027AAP | Argentina |
| 1107 | Comite Independiente de Etica para Ensayos en Famacologia Clinica | Instituto Donato | Buenos Aires | Ciudad Autonoma Buenos Aires | C1027AAP | Argentina |
| 1108 | Comite Independiente de Etica para Ensayos en Famacologia Clinica | Instituto de la Visión | Buenos Aires | Ciudad Autonoma Buenos Aires | C1027AAP | Argentina |
| 1109 | Comité de Ética en Investigación Instituto de Investigaciones Clínicas | *Oftalmologia Integral* | Mar del Plata | Buenos Aires | B7600FZN | Argentina |
| 1110 | Comité de Ética en Investigación Instituto de Investigaciones Clínicas | Clinica Modelo de Lanus | Buenos Aires | Buenos Aires | C1027AAP | Argentina |
| 1111 | Comite Independiente de Etica para Ensayos en Famacologia Clinica | Centro Oftalmologico Ventola | Buenos Aires | Buenos Aires | C1027AAP | Argentina |
| 1113 | Comite Independiente de Etica para Ensayos en Famacologia Clinica | Clínica Dr Rivero Covre | Buenos Aires | Santa Fe | C1027AAP | Argentina |
| 1114 | Comite Independiente de Etica Fundacion Rusculleda | Instituto Oftamológico de Córdoba SA | Cordoba | Cordoba | X5003DC E | Argentina |
| 1115 | Comite de Etica de CER Investigaciones Clinicas (CECIC) | Hospital Oftalmológico Malvinas Argentinas | Quilmes | Buenos Aires | B1878DVB | Argentina |
| 1116 | Comite Independiente de Etica para Ensayos en Famacologia Clinica | Consultorios Oftalmologico Dres Fugazzotto | Buenos Aires | Mendoza | C1027AAP | Argentina |
| 1117 | Comite Independiente de Etica para Ensayos en Famacologia Clinica | Plaza Vision S.A. | Buenos Aires | Mendoza | C1027AAP | Argentina |

| **Center Number** | **Ethics Committee or Institutional Review Board** | **Department / Organization** | **EC/IRB**  **City** | **EC/IRB**  **State/ Province** | **EC/IRB**  **Postal Code** | **Center Country** |
| --- | --- | --- | --- | --- | --- | --- |
| 1118 | Comite Independiente de Etica para Ensayos en Famacologia Clinica | Grupo laser Visión - Rosario Eximer Laser Visión | Buenos Aires | Santa Fe | C1027AAP | Argentina |
| 1119 | Comite de Etica de CER Investigaciones Clinicas (CECIC) | Clínica Oftalmológica Meroni | Quilmes | Buenos Aires | B1878DVB | Argentina |
| 3001 | Ethik-Kommision Der Medizinischen Universitat Wien | General University Hospital of Vienna | Wien | Vienna | 1090 | Austria |
| 3002 | Ethik-Kommision Der Medizinischen Universitat Wien | Prof. Dr. Siegfried Priglinger | Wien | Vienna | 1090 | Austria |
| 2000 | NSW Government Health Sydney Local Health District | Sydney Eye Hospital, Research Development Office | Camperdo wn | New South Wales | 2050 | Australia |
| 2001 | Bellberry Human Research Ethics Committee | Lions Eye Institute | North Adelaide | South Australia | 5065 | Australia |
| 2002 | Human Research Ethics Committee (Tasmania) Network | Hobart Eye Surgeions, University of Tasmania | Hobart | Tasmania | 7001 | Australia |
| 2003 | Bellberry Human Research Ethics Committee | Eye Consultants SA | North Adelaide | South Australia | 5065 | Australia |
| 2004 | Bellberry Human Research Ethics Committee | Vision Eye Institute Chatswood | North Adelaide | South Australia | 5065 | Australia |
| 2005 | Tasmania Health and Medical Human Research Ethics Committee | Office of Research Services, University of Tasmania | Hobart | Tasmania | 7001 | Australia |
| 2006 | Bellberry Human Research Ethics Committee | Retina & Vitreous Centre | North Adelaide | New South Wales | 5065 | Australia |
| 2007 | Bellberry Human Research Ethics Committee | Vision Retinal Institute | North Adelaide | Queenslan d | 5065 | Australia |
| 2008 | Bellberry Human Research Ethics Committee | Forster Eye Surgery | North Adelaide | New South Wales | 5065 | Australia |
| 2010 | The Royal Victorian Eye & Ear Hospital | Human Research & Ethics Committee | East Melbourne | Victoria | 8002 | Australia |
| 2011 | Bellberry Human Research Ethics Committee | Brisbane Eye Clinic | North Adelaide | Queenslan d | 5065 | Australia |
| 2012 | Bellberry Human Research Ethics Committee | Sydney Retina Eye Clinic and Day Surgery | North Adelaide | New South Wales | 5065 | Australia |
| 2013 | Bellberry Human Research Ethics Committee | Macquarie University | North Adelaide | New South Wales | 5064 | Australia |
| 2014 | Bellberry Human Research Ethics Committee | Marsden Eye Specialists | North Adelaide | New South Wales | 5065 | Australia |
| 2017 | Bellberry Human Research Ethics Committee | Southern Ophthalmology | North Adelaide | New South Wales | 5065 | Australia |
| 2018 | Bellberry Human Research Ethics Committee | Strathfield Retina Clinic | North Adelaide | New South Wales | 5065 | Australia |
| 2019 | Bellberry Human Research Ethics Committee | Retina Associates - Chatswood Retina Service | North Adelaide | New South Wales | 5065 | Australia |
| 2020 | Bellberry Human Research Ethics Committee | Macquarie University | North Adelaide | New South Wales | 5065 | Australia |
| 2021 | Bellberry Human Research Ethics Committee | Adelaide Eye and Retina Centre | North Adelaide | South Australia | 5065 | Australia |
| 2024 | Bellberry Human Research Ethics Committee | Queensland Eye Institute | North Adelaide | Queenslan d | 5065 | Australia |

| **Center Number** | **Ethics Committee or Institutional Review Board** | **Department / Organization** | **EC/IRB**  **City** | **EC/IRB**  **State/ Province** | **EC/IRB**  **Postal Code** | **Center Country** |
| --- | --- | --- | --- | --- | --- | --- |
| 2025 | Bellberry Human Research Ethics Committee | Melbourne Retina Associates | North Adelaide | Victoria | 5065 | Australia |
| 2026 | Bellberry Human Research Ethics Committee | Private Rooms, Eye Clinic (B4a) Westmead Hospital | North Adelaide | New South Wales | 5065 | Australia |
| 2032 | Bellberry Human Research Ethics Committee | St. John of God Hospital | North Adelaide | Victoria | 5065 | Australia |
| 2034 | Bellberry Human Research Ethics Committee | Oakleigh Eye Center | North Adelaide | Victoria | 5065 | Australia |
| 2035 | Bellberry Human Research Ethics Committee | Retina and Vitreous Centre | North Adelaide | New South Wales | 5065 | Australia |
| 2037 | Bellberry Human Research Ethics Committee | Waverley Eye Clinic | North Adelaide | Victoria | 5065 | Australia |
| 3100 | Committee for Medical Ethics/Clinical Research | Faculty of Medicine UZ Gathuisberg | Leuven |  | 3000 | Belgium |
| 3101 | Committee for Medical Ethics/Clinical Research | Faculty of Medicine UZ Gathuisberg | Leuven | Hasselt | 3000 | Belgium |
| 3102 | Committee for Medical Ethics/Clinical Research | Faculty of Medicine UZ Gathuisberg | Leuven |  | 3000 | Belgium |
| 3104 | Committee for Medical Ethics/Clinical Research | Faculty of Medicine UZ Gathuisberg | Leuven |  | 3000 | Belgium |
| 3106 | Committee for Medical Ethics/Clinical Research | Faculty of Medicine UZ Gathuisberg | Leuven |  | 3000 | Belgium |
| 3107 | Committee for Medical Ethics/Clinical Research | Faculty of Medicine UZ Gathuisberg | Leuven |  | 3000 | Belgium |
| 3110 | Committee for Medical Ethics/Clinical Research | Faculty of Medicine UZ Gathuisberg | Leuven |  | 3000 | Belgium |
| 3111 | Committee for Medical Ethics/Clinical Research | Faculty of Medicine UZ Gathuisberg | Leuven |  | 3000 | Belgium |
| 3112 | Committee for Medical Ethics/Clinical Research | Faculty of Medicine UZ Gathuisberg | Leuven |  | 3000 | Belgium |
| 3113 | Comite d'Ethique Centre Hospitalier de Mouscron | Centre Hospitalier de Mouscron | Mouscron |  | 7700 | Belgium |
| 3114 | Committee for Medical Ethics/Clinical Research | Faculty of Medicine UZ Gathuisberg | Leuven |  | 3000 | Belgium |
| 3117 | Committee for Medical Ethics/Clinical Research | Faculty of Medicine UZ Gathuisberg | Leuven |  | 3000 | Belgium |
| 3118 | Committee for Medical Ethics/Clinical Research | Faculty of Medicine UZ Gathuisberg | Leuven |  | 3000 | Belgium |
| 3120 | Committee for Medical Ethics/Clinical Research | Faculty of Medicine UZ Gathuisberg | Leuven |  | 3000 | Belgium |
| 1200 | Hospital Municipal Dr. Mario Gatti | Centro Medico de Oftalmologia | Cambui | Campinas | 13092 | Brazil |
| 1203 | Comissao Nacional de Etica Em Pesquisa | Instituto da Visão | Belo Horizonte | Minas Gerais | 30150 | Brazil |
| 1204 | Comissao Nacional de Etica Em Pesquisa | HFSE - Hospital Federal dos Servidores do Estado do Rio de Janeiro | Belo Horizonte | Rio de Janeiro | 30150 | Brazil |
| 1205 | Comissao Nacional de Etica Em Pesquisa | CBCO - Centro Brasileiro de Cirurgia de Olhos | Belo Horizonte | Goiás | 30150 | Brazil |
| 1208 | Comissao Nacional de Etica Em Pesquisa | ANGIOCORPORE | Belo Horizonte | Sao Paulo | 30150 | Brazil |
| 1209 | Comissao Nacional de Etica Em Pesquisa | Clínica Lavinsky Oftalmologia | Belo Horizonte | Rio Grande do Sul | 30150 | Brazil |
| 1000 | Ontario IRB/REB | IRB Services | Aurora | New Brunswick | L4G 0A5 | Canada |

| **Center Number** | **Ethics Committee or Institutional Review Board** | **Department / Organization** | **EC/IRB**  **City** | **EC/IRB**  **State/ Province** | **EC/IRB**  **Postal Code** | **Center Country** |
| --- | --- | --- | --- | --- | --- | --- |
| 1001 | Ontario IRB/REB | IRB Services | Aurora | Nova Scotia | L4G 0A5 | Canada |
| 1003 | Ontario IRB/REB | IRB Services | Aurora | Ontario | L4G 0A5 | Canada |
| 1008 | Ontario IRB/REB | IRB Services | Aurora | Ontario | L4G 0A5 | Canada |
| 1007 | University Health Network | Research Ethics Board | Toronto | Ontario | M5G 1Z5 | Canada |
| 1006 | Ontario IRB/REB | IRB Services | Aurora | Ontario | L4G 0A5 | Canada |
| 1005 | Ontario IRB/REB | IRB Services | Aurora | Ontario | L4G 0A5 | Canada |
| 1004 | Ontario IRB/REB | IRB Services | Aurora | Quebec | L4G 0A5 | Canada |
| 1010 | Ontario IRB/REB | IRB Services | Aurora | Quebec | L4G 0A5 | Canada |
| 1011 | Ottawa Health Science Network Research Ethics Board | The Ottawa Hospital  - General Campus, University of Ottawa Eye Institute | Ottawa | Ontario | K1Y 4E9 | Canada |
| 1012 | Ontario IRB/REB | The Retina Centre of Ottawa | Aurora | Ontario | L4G 0A5 | Canada |
| 1013 | Ontario IRB/REB | IRB Services | Aurora | Ontario | L4G 0A5 | Canada |
| 1014 | Ontario IRB/REB | IRB Services | Aurora | British Columbia | L4G 0A5 | Canada |
| 1015 | Health Research Ethics Board of Alberta | IRB Services | Edmonton | Alberta | T5J 4A7 | Canada |
| 1016 | Ontario IRB/REB | Clincial Trials Committee | Aurora | Ontario | L4G 0A5 | Canada |
| 1018 | Ontario IRB/REB | IRB Services | Aurora | Ontario | L4G 0A5 | Canada |
| 1020 | Ontario IRB/REB | IRB Services | Aurora | Ontario | L4G 0A5 | Canada |
| 1021 | Ontario IRB/REB | IRB Services | Aurora | Ontario | L4G 0A5 | Canada |
| 1019 | Ontario IRB/REB | IRB Services | Aurora | Ontario | L4G 0A5 | Canada |
| 8200 | Resolucion Comite Etico Cientifico | Hospital del Cobre Salvador Allende Gossens | La Serena |  | 1399001 | Chile |
| 2101 | Beijing Tongren Hospital EC | Renmin Hospital of Wuhan University | Wuhan | Hubei | 430060 | China |
| 2102 | Beijing Tongren Hospital EC | No. 10 People's Hospital of Shanghai |  | Shanghai | 200072 | China |
| 2104 | Beijing Tongren Hospital EC | Eye and ENT hospital |  | Shanghai | 200000 | China |
| 2106 | Beijing Tongren Hospital EC | Zhongshan Ophthalmic Center, Sun Yat-sen University | Guangzho u | Guangdon g | 510060 | China |
| 2107 | Beijing Tongren Hospital EC | Peking University First Hospital | Beijing,P. R. | Beijing | 100034 | China |
| 2108 | Beijing Tongren Hospital EC | Tianjin Medical University Eye Center | Tianjin |  | 300384 | China |
| 2109 | Beijing Tongren Hospital EC | Xinhua Hospital Affiliated to Shanghai Jiao Tong University School of Medicine |  | Shanghai | 200092 | China |
| 2100 | Beijing Tongren Hospital EC | Beijing Tong Ren Hospital, Capital Medical University |  | Beijing | 100730 | China |
| 2115 | Beijing Tongren Hospital EC | Peking University Third Hospital |  | Beijing | 100191 | China |
| 2116 | Beijing Tongren Hospital EC | Chinese PLA General Hospital | Beijing | Beijing | 100853 | China |
| 2117 | Beijing Tongren Hospital EC | Beijing Hospital |  | Beijing | 100730 | China |
| 2119 | Beijing Tongren Hospital EC | Peking Union Medical College Hospital |  | Beijing | 100032 | China |
| 2121 | Beijing Tongren Hospital EC | Southwest Hospital | Chongqing | Chongqing | 400038 | China |
| 2122 | Beijing Tongren Hospital EC | Shanghai First People's Hospital |  | Shangai | 200080 | China |
| 2124 | Beijing Tongren Hospital EC | Xiamen Eye Centre | Fujian |  | 361001 | China |

| **Center Number** | **Ethics Committee or Institutional Review Board** | **Department / Organization** | **EC/IRB**  **City** | **EC/IRB**  **State/ Province** | **EC/IRB**  **Postal Code** | **Center Country** |
| --- | --- | --- | --- | --- | --- | --- |
| 2127 | Beijing Tongren Hospital EC | Shierming Eye Hospital | Jinan | Shandong | 250001 | China |
| 2128 | Beijing Tongren Hospital EC | The Affiliated Hospital of Guiyang Medical College | Guiyang | Guizhou | 500000 | China |
| 2126 | Beijing Tongren Hospital EC | No. 474 Hospital of PLA | Wulumuqi | Xinjiang Uygur | 830000 | China |
| 2130 | Beijing Tongren Hospital EC | No.2 Hospital Affiliated to Jilin University | Changchu n City | Jilin | 130041 | China |
| 2129 | Beijing Tongren Hospital EC | Yunnan 2nd People's Hospital | Kunming | Yun'nan | 650021 | China |
| 1304 | Sociedad de Cirugia Ocular S.A. | Sociedad de Cirugía Ocular | Bogota |  |  | Colombia |
| 1302 | Instituto para Ninos Ciegos y Sordos del Valle del Cauca | Instituto de Ciegos y Sordos INSORP | San Fernando | Cali |  | Colombia |
| 1306 | Sociedad de Cirugia Ocular S.A. | Clínica Barraquer | Bogota |  |  | Colombia |
| 1307 | Sociedad de Cirugia Ocular S.A. | Clinica Oftalmologica | Bogota |  |  | Colombia |
| 1309 | Comite de Etica en la Investigacion | OPTISALUD SAS | Bogota |  |  | Colombia |
| 1308 | Sociedad de Cirugia Ocular S.A. | Clínica Oftalmológica del Atlántico | Bogota |  |  | Colombia |
| 3300 | Eticka komise Fakultni nemocnice Hradec Kralove | Fakultni nemocnice Hradec Kralove | Hradec Kralove |  | 500 05 | Czech Republic |
| 3302 | Eticka komise Fakultni Nemocnice Kralovske Vinohrady | Fakultni nemocnice Kralovske Vinohrady | Praha |  | 100 34 | Czech Republic |
| 3303 | Eticka komise FN Ostrava | University Hopsital Ostrava | Ostrava- Poruba |  | 70800 | Czech Republic |
| 3305 | Eticka komise Vseobecne fakultni nemocnice v Praze | Charles University Hopsital 1st Faculty of Medicine | Praha 2 | Praha 2 | 128 08 | Czech Republic |
| 3307 | Eticka komise Fakultni Nemocnice Brno | Fakultni nemocnice Brno | Brno |  | 625 00 | Czech Republic |
| 3306 | Eticka komise Ustredni vojenske nemocnice | Ustredni vojenska nemocnice Praha | Praha 6 |  | 169 02 | Czech Republic |
| 3301 | Eticka komise-Krajska zdravotni a.s. | Krajska zdravotni,  a.s. - Masarykova nemocnice v Usti nad Labem | Usti nad Labem |  | 40113 | Czech Republic |
| 3308 | Eticka komise FN a LF UP Olomouc-LEC | Fakultni nemocnice Olomouc | Olomouc |  | 775 20 | Czech Republic |
| 3304 | Eticka komise Fakultni nemocnice Plzen | Fakultni nemocnice Plzen | Plzen |  | 305 99 | Czech Republic |
| 3600 | Ethikkommission an der Medizinischen Fakultaet der Rheinischen Friedrich-Wilhelms- Universitaet Bonn | University of Bonn | Bonn | Nordrhein Westfalen | 53105 | Germany |
| 3601 | Aerztekammer Berlin | Praxis Arzt für Augenheilkunde | Berlin |  | 10969 | Germany |
| 3604 | Ethik-Kommission der Ärztekammer Westfalen- Lippe und der Med.  Fakultät der Universität Münster | St. Franziskus Hospital | Muenster | Nordrhein Westfalen | 48147 | Germany |
| 3602 | Ethikkommission an der Medizinischen Fakultaet der Rheinischen Friedrich-Wilhelms- Universitaet Bonn | Augenarztpraxis Grasbon | Bonn | Bayern | 53105 | Germany |
| 3606 | Aerztekammer Berlin | Praxis Steinberg | Berlin |  | 10969 | Germany |
| 3607 | Ethik-Kommission der Ärztekammer Westfalen- Lippe und der Med.  Fakultät der Universität Münster | Universitaetsklinikum Muenster | Muenster | Nordrhein Westfalen | 48147 | Germany |

| **Center Number** | **Ethics Committee or Institutional Review Board** | **Department / Organization** | **EC/IRB**  **City** | **EC/IRB**  **State/ Province** | **EC/IRB**  **Postal Code** | **Center Country** |
| --- | --- | --- | --- | --- | --- | --- |
| 3608 | Ethikkommission der erztekammer Hamburg | Praxis_Dr Kaupke | Hamburg |  | 22083 | Germany |
| 3609 | Ethik-Kommission der Ärztekammer Westfalen- Lippe und der Med.  Fakultät der Universität Münster | Klinikum Lüdenscheid | Muenster | Nordrhein Westfalen | 48147 | Germany |
| 3611 | Landesaerztekammer Baden-Wuerttemberg | Dr. Rabethge Klinik GmbH | Stuttgart | Baden Wuerttem berg | 70597 | Germany |
| 3612 | Universitaetsklinikum Tuebingen | Eberhard Karls University Eye Hospital | Tuebingen | Baden Wuerttem berg | 72074 | Germany |
| 3615 | Ethik-Kommission der Aerztekammer Niedersachsen | Klinikum Osnabrück | Hannover | Niedersac hsen | 30175 | Germany |
| 3616 | Geschaeftsstelle der Ethikkommission | Universitaetsklinikum Koeln | Koeln | Nordrhein Westfalen | 50931 | Germany |
| 3628 | Augenlaserzentrum Neu- Ulm | Augenlaserzentrum | Neu-Ulm | Bayern | 89231 | Germany |
| 3629 | Ethik-Kommission der Aerztekammer Niedersachsen | Augenklinik Dannenberg | Hannover | Niedersac hsen | 30175 | Germany |
| 3632 | Ethik-Kommission der Ärztekammer Westfalen- Lippe und der Westfälischen Wilhelms- Universität Münster | Private practice_ Dr Grote-Schmidt | Münster | Nordrhein Westfalen | 48147 | Germany |
| 3631 | An die Ethik-Kommission der Bayerischen Landesärztekammer | Fachärztin für Augenheilkunde | Munich | Bayern | 81677 | Germany |
| 3633 | An die Ethik-Kommission der Bayerischen Landesärztekammer | Klinikum Augsburg Augenklinik | Munich | Bayern | 81677 | Germany |
| 3630 | An die Ethik-Kommission der Bayerischen Landesärztekammer | Dr. med. Claus Fuchs Fachärzte für Augenheilkunde | Munich | Bayern | 81677 | Germany |
| 8300 | Comite de Bioetica de la Universidad Central del Ecuador (COBI-UCE) | Centro Medico Quirurgico Oftalmologico Alta Vision | Quito |  | 593 | Ecuador |
| 4600 | CEIC Hospital Universitario Ramon y Cajal | Vissum Corporación Oftalmológica – Mirassierra | Madrid |  | 28034 | Spain |
| 4601 | CEIC Hospital Universitario Ramon y Cajal | Hospital Clinico Universitario Lozano Blesa | Madrid |  | 28035 | Spain |
| 4602 | CEIC Hospital Universitario Ramon y Cajal | Hospital Universitari i Politecnic La Fe | Madrid |  | 28036 | Spain |
| 4604 | CEIC Hospital Universitario Ramon y Cajal | Hospital Universitario Clinico San Carlos | Madrid |  | 28039 | Spain |
| 4605 | CEIC Hospital Universitario Ramon y Cajal | Hospital Universitario 12 de Octubre | Madrid |  | 28040 | Spain |
| 4606 | CEIC Hospital Universitario Ramon y Cajal | Hospital Universitario Miguel Servet | Madrid |  | 28043 | Spain |
| 4608 | CEIC Hospital Universitario Ramon y Cajal | Hospital de la Santa Creu i Sant Pau | Madrid |  | 28045 | Spain |
| 4612 | CEIC Hospital Universitario Ramon y Cajal | Universidad de Valladolid | Madrid |  | 28049 | Spain |
| 4614 | CEIC Hospital Universitario Ramon y Cajal | Hospital General de Catalunya | Madrid | Barcelona | 28049 | Spain |

| **Center Number** | **Ethics Committee or Institutional Review Board** | **Department / Organization** | **EC/IRB**  **City** | **EC/IRB**  **State/ Province** | **EC/IRB**  **Postal Code** | **Center Country** |
| --- | --- | --- | --- | --- | --- | --- |
| 4615 | CEIC Hospital Universitario Ramon y Cajal | Hospital San Pedro | Madrid | La Rioja | 28049 | Spain |
| 4620 | CEIC Hospital Universitario Ramon y Cajal | Hospital Universitario Principe de Asturias | Madrid | Madrid | 28049 | Spain |
| 4622 | CEIC Hospital Universitario Ramon y Cajal | Hospital Universitario de Burgos | Madrid |  | 28049 | Spain |
| 4623 | CEIC Hospital Universitario Ramon y Cajal | Hospital Universitario de Salamanca | Madrid |  | 28049 | Spain |
| 4624 | CEIC Hospital Universitario Ramon y Cajal | Hospital Moncloa | Madrid |  | 28049 | Spain |
| 4625 | CEIC Hospital Universitario Ramon y Cajal | Hospital Universitario de Leon | Madrid |  | 28049 | Spain |
| 4626 | CEIC Hospital Universitario Ramon y Cajal | Hospital Universitario Lucus Augusti | Madrid |  | 28049 | Spain |
| 4627 | CEIC Hospital Universitario Ramon y Cajal | Hospital Dos de Maig | Madrid | Barcelona | 28050 | Spain |
| 4628 | CEIC Hospital Universitario Ramon y Cajal | Hospital Arnau de Vilanova | Madrid |  | 28050 | Spain |
| 4630 | CEIC Hospital Universitario Ramon y Cajal | Clinica Rementeria | Madrid |  | 28051 | Spain |
| 4634 | CEIC Hospital Universitario Ramon y Cajal | Centro de Oftalmología Barraquer | Madrid |  | 28052 | Spain |
| 4632 | CEIC Hospital Universitario Ramon y Cajal | Hospital Regional Universitario de Malaga | Madrid |  | 28053 | Spain |
| 4631 | CEIC Hospital Universitario Ramon y Cajal | Hospital Universitario Sant Joan de Reus | Madrid | Tarragona | 28054 | Spain |
| 4629 | CEIC Hospital Universitario Ramon y Cajal | Fundacio Privada Hospital Asil de Granollers | Madrid | Barcelona | 28055 | Spain |
| 4633 | CEIC Hospital Universitario Ramon y Cajal | Centro Oftalmologico Gaztambide | Madrid |  | 28056 | Spain |
| 3500 | N/A | Centre Hospitalier Intercommunal de Créteil | Creteil | Val de Marne |  | France |
| 3501 | N/A | Centre Ophtalmologique d’Imagerie et de Laser | Paris | Val de Marne | 94010 | France |
| 3502 | N/A | Clinique Ocean | Vannes | Côte-d'Or | 75015 | France |
| 3503 | N/A | CHU Dijon - Hopital General | Dijon | Côte-d'Or | 56000 | France |
| 3504 | N/A | Visiopole Private Practice | Lagord | Charente | 21000 | France |
| 3505 | N/A | CHU de Nice - Hôpital Lenval | Nice | Alpes Maritimes | 17140 | France |
| 3506 | N/A | Cabinet Odeon | Paris |  | 060 06 | France |
| 3507 | N/A | Groupe Hospitalier Pellegrin -Hôpital Pellegrin | Bordeaux | Gironde | 75006 | France |
| 3518 | N/A | Centre Ophtalmologique de L'Odeon | Paris |  | 33000 | France |
| 3508 | N/A | Hôpital Lariboisière | Paris |  | 75006 | France |
| 3509 | N/A | Polyclinique de Courlancy | Reims | Marne | 75475 | France |
| 3510 | N/A | Clinique Orl Honore Cave | Montauba n | Tarn et Garonne | 51100 | France |
| 3511 | N/A | Cabinet d'Ophtalmologie | Melun | Seine et Marne | 82000 | France |

| **Center Number** | **Ethics Committee or Institutional Review Board** | **Department / Organization** | **EC/IRB**  **City** | **EC/IRB**  **State/ Province** | **EC/IRB**  **Postal Code** | **Center Country** |
| --- | --- | --- | --- | --- | --- | --- |
| 3512 | N/A | CHU Nantes - Hôtel Dieu | Nantes Cedex 1 | Loire Atlantique | 77000 | France |
| 3513 | N/A | Centre Hospitalier de la Croix Rousse | Lyon | Rhone | 44093 | France |
| 3514 | N/A | CHU Toulouse, Hôpital Paule de Vignier | Toulouse | Cedex 9 | 69317 | France |
| 3516 | N/A | Fondation Ophtalmologique Adolphe de Rothschild | Paris |  | 31059 | France |
| 3517 | N/A | Clinique de Montargis | Montargis | Loiret | 75019 | France |
| 5000 | NRES Committee Yorkshire and the Humber Leeds East | Central Ethics Committee | Jarrow | Lancashir e | 45200 | United Kingdom |
| 5001 | NRES Committee Yorkshire and the Humber Leeds East | Central Ethics Committee | Jarrow | South Yorkshire | NE 32 3DT | United Kingdom |
| 5002 | NRES Committee Yorkshire and the Humber Leeds East | Central Ethics Committee | Jarrow | Tyne & Wear | NE 32 3DT | United Kingdom |
| 5003 | NRES Committee Yorkshire and the Humber Leeds East | Central Ethics Committee | Jarrow |  | NE 32 3DT | United Kingdom |
| 5004 | NRES Committee Yorkshire and the Humber Leeds East | Central Ethics Committee | Jarrow | Surrey | NE 32 3DT | United Kingdom |
| 5005 | NRES Committee Yorkshire and the Humber Leeds East | Central Ethics Committee | Jarrow | North Yorkshire | NE 32 3DT | United Kingdom |
| 5006 | NRES Committee Yorkshire and the Humber Leeds East | Central Ethics Committee | Jarrow | Greater Manchest er | NE 32 3DT | United Kingdom |
| 5009 | NRES Committee Yorkshire and the Humber Leeds East | Central Ethics Committee | Jarrow |  | NE 32 3DT | United Kingdom |
| 5030 | NRES Committee Yorkshire and the Humber Leeds East | Central Ethics Committee | Jarrow | Greater London | NE 32 3DT | United Kingdom |
| 5010 | NRES Committee Yorkshire and the Humber Leeds East | Central Ethics Committee | Jarrow | Norfolk | NE 32 3DT | United Kingdom |
| 5011 | NRES Committee Yorkshire and the Humber Leeds East | Central Ethics Committee | Jarrow | Staffordshi re | NE 32 3DT | United Kingdom |
| 5012 | NRES Committee Yorkshire and the Humber Leeds East | Central Ethics Committee | Jarrow |  | NE 32 3DT | United Kingdom |
| 5013 | NRES Committee Yorkshire and the Humber Leeds East | Central Ethics Committee | Jarrow | West Midlands | NE 32 3DT | United Kingdom |
| 5014 | NRES Committee Yorkshire and the Humber Leeds East | Central Ethics Committee | Jarrow | West Midlands | NE 32 3DT | United Kingdom |
| 5015 | NRES Committee Yorkshire and the Humber Leeds East | Central Ethics Committee | Jarrow | West Yorkshire | NE 32 3DT | United Kingdom |
| 5024 | NRES Committee Yorkshire and the Humber Leeds East | Central Ethics Committee | Jarrow | Surrey | NE 32 3DT | United Kingdom |
| 5055 | NRES Committee Yorkshire and the Humber Leeds East | Central Ethics Committee | Jarrow | Greater London | NE 32 3DT | United Kingdom |
| 5036 | NRES Committee Yorkshire and the Humber Leeds East | Central Ethics Committee | Jarrow | Gloucester shire | NE 32 3DT | United Kingdom |
| 5037 | NRES Committee Yorkshire and the Humber Leeds East | Central Ethics Committee | Jarrow | Greater London | NE 32 3DT | United Kingdom |
| 5016 | NRES Committee Yorkshire and the Humber Leeds East | Central Ethics Committee | Jarrow | West Midlands | NE 32 3DT | United Kingdom |

| **Center Number** | **Ethics Committee or Institutional Review Board** | **Department / Organization** | **EC/IRB**  **City** | **EC/IRB**  **State/ Province** | **EC/IRB**  **Postal Code** | **Center Country** |
| --- | --- | --- | --- | --- | --- | --- |
| 5029 | NRES Committee Yorkshire and the Humber Leeds East | Central Ethics Committee | Jarrow | Hampshire | NE 32 3DT | United Kingdom |
| 5021 | NRES Committee Yorkshire and the Humber Leeds East | Central Ethics Committee | Jarrow |  | NE 32 3DT | United Kingdom |
| 5017 | NRES Committee Yorkshire and the Humber Leeds East | Central Ethics Committee | Jarrow | Hampshire | NE 32 3DT | United Kingdom |
| 5022 | NRES Committee Yorkshire and the Humber Leeds East | Central Ethics Committee | Jarrow | Cheshire | NE 32 3DT | United Kingdom |
| 5018 | NRES Committee Yorkshire and the Humber Leeds East | Central Ethics Committee | Jarrow | Greater London | NE 32 3DT | United Kingdom |
| 5019 | NRES Committee Yorkshire and the Humber Leeds East | Central Ethics Committee | Jarrow | North Yorkshire | NE 32 3DT | United Kingdom |
| 5020 | NRES Committee Yorkshire and the Humber Leeds East | Central Ethics Committee | Jarrow | North Yorkshire | NE 32 3DT | United Kingdom |
| 5026 | NRES Committee Yorkshire and the Humber Leeds East | Central Ethics Committee | Jarrow | West Midlands | NE 32 3DT | United Kingdom |
| 5025 | NRES Committee Yorkshire and the Humber Leeds East | Central Ethics Committee | Jarrow | West Midlands | NE 32 3DT | United Kingdom |
| 5044 | NRES Committee Yorkshire and the Humber Leeds East | Central Ethics Committee | Jarrow | Leicesters hire | NE 32 3DT | United Kingdom |
| 5031 | NRES Committee Yorkshire and the Humber Leeds East | Central Ethics Committee | Jarrow | Isle of Wight | NE 32 3DT | United Kingdom |
| 5032 | NRES Committee Yorkshire and the Humber Leeds East | Central Ethics Committee | Jarrow | Lancashir e | NE 32 3DT | United Kingdom |
| 5023 | NRES Committee Yorkshire and the Humber Leeds East | Central Ethics Committee | Jarrow | West Yorkshire | NE 32 3DT | United Kingdom |
| 5028 | NRES Committee Yorkshire and the Humber Leeds East | Central Ethics Committee | Jarrow | Greater London | NE 32 3DT | United Kingdom |
| 5033 | NRES Committee Yorkshire and the Humber Leeds East | Central Ethics Committee | Jarrow | Greater London | NE 32 3DT | United Kingdom |
| 5038 | NRES Committee Yorkshire and the Humber Leeds East | Central Ethics Committee | Jarrow | Oxfordshir e | NE 32 3DT | United Kingdom |
| 5027 | NRES Committee Yorkshire and the Humber Leeds East | Central Ethics Committee | Jarrow | Essex | NE 32 3DT | United Kingdom |
| 5039 | NRES Committee Yorkshire and the Humber Leeds East | Central Ethics Committee | Jarrow | Greater London | NE 32 3DT | United Kingdom |
| 5034 | NRES Committee Yorkshire and the Humber Leeds East | Central Ethics Committee | Jarrow | Merseysid e | NE 32 3DT | United Kingdom |
| 5040 | NRES Committee Yorkshire and the Humber Leeds East | Central Ethics Committee | Jarrow | Devon | NE 32 3DT | United Kingdom |
| 5041 | NRES Committee Yorkshire and the Humber Leeds East | Central Ethics Committee | Jarrow | Highland Region | NE 32 3DT | United Kingdom |
| 5042 | NRES Committee Yorkshire and the Humber Leeds East | Central Ethics Committee | Jarrow |  | NE 32 3DT | United Kingdom |
| 5047 | NRES Committee Yorkshire and the Humber Leeds East | Central Ethics Committee | Jarrow | Norfolk | NE 32 3DT | United Kingdom |
| 5035 | NRES Committee Yorkshire and the Humber Leeds East | Central Ethics Committee | Jarrow | Avon | NE 32 3DT | United Kingdom |

| **Center Number** | **Ethics Committee or Institutional Review Board** | **Department / Organization** | **EC/IRB**  **City** | **EC/IRB**  **State/ Province** | **EC/IRB**  **Postal Code** | **Center Country** |
| --- | --- | --- | --- | --- | --- | --- |
| 5043 | NRES Committee Yorkshire and the Humber Leeds East | Central Ethics Committee | Jarrow | Kent | NE 32 3DT | United Kingdom |
| 5046 | NRES Committee Yorkshire and the Humber Leeds East | Central Ethics Committee | Jarrow | Derbyshire | NE 32 3DT | United Kingdom |
| 5060 | NRES Committee Yorkshire and the Humber Leeds East | Central Ethics Committee | Jarrow | Bedfordshi re | NE 32 3DT | United Kingdom |
| 5061 | NRES Committee Yorkshire and the Humber Leeds East | Central Ethics Committee | Jarrow | Merseysid e | NE 32 3DT | United Kingdom |
| 5062 | NRES Committee Yorkshire and the Humber Leeds East | Central Ethics Committee | Jarrow | Worcester shire | NE 32 3DT | United Kingdom |
| 3700 | Scientific Council of University Eye Hospital of Haraklion | University Eye Hospital of Heraklion | Heraklion |  | 71201 | Greece |
| 3701 | Scientific Council of Eye Hospital of Athens | Eye Hospital of Athens -1st Clinic | Athens |  | 10672 | Greece |
| 3702 | Scientific Council of Omma Opthalmological Institute of Athens | Omma Ophtalmological Institute of Athens | Athens |  | 11525 | Greece |
| 3703 | Scientific Council of General Hospital Papageorgiou | General Hospital Papageorgiou | Thessaloni ki |  | 56429 | Greece |
| 3704 | Scientific Council of Red Cross Hospital | Red Cross Hospital - 2nd Clinic | Athens |  | 11526 | Greece |
| 3705 | Scientific Council of University Hospital of Alexandroupolis | University General Hospital of Alexandroupolis | Alexandro upolis |  | 68100 | Greece |
| 3710 | Ethics Committee of University Hospital of Larissa | University General Hospital of Larissa | Mezourlo |  | 41110 | Greece |
| 3712 | Scientific Council of Athens Vision Eye Institute | Athens Vision Eye Institute | Athens |  | 17673 | Greece |
| 3714 | Scientific Council of Athens Eye Hospital | Ophtalmiatrio Athinon - Athens Eye Hospital | Athens |  | 10672 | Greece |
| 3715 | Ethics Committee of General Hospital of Lamia | General Hospital of Lamia | Lamia |  | 35100 | Greece |
| 3716 | Ethics Committee of University Hospital of Patra | University Hopsital of Patras | Patra |  | 26504 | Greece |
| 3717 | Ethics Committee of General Hospital of Rethymnon | General Hospital of Rethymnon | Rethymno n |  | 74100 | Greece |
| 8800 | Research Ethics Committee (Kowloon Central/Kowloon East) | Hong Kong Eye Hospital | Kowloon |  | N/A | Hong Kong |
| 3800 | Egeszsegugyi Tudomanyos Tanacs Tudomanyos es Kutatasetikai Bizottsag | Budapest Retina Associates | Budapest |  | 1051 | Hungary |
| 3802 | Egeszsegugyi Tudomanyos Tanacs Tudomanyos es Kutatasetikai Bizottsag | Semmelweis Egyetem | Budapest |  | 1051 | Hungary |
| 3801 | Egeszsegugyi Tudomanyos Tanacs Tudomanyos es Kutatasetikai Bizottsag | Debreceni Egyetem Klinikai Kozpont | Budapest |  | 1051 | Hungary |
| 3803 | Egeszsegugyi Tudomanyos Tanacs Tudomanyos es Kutatasetikai Bizottsag | Pecsi Tudomanyegyetem | Budapest |  | 1051 | Hungary |
| 3804 | Egeszsegugyi Tudomanyos Tanacs Tudomanyos es Kutatasetikai Bizottsag | Szegedi Tudomanyegyetem Szent-Gyorgyi Albert Klinikai Kozpont | Budapest |  | 1051 | Hungary |

| **Center Number** | **Ethics Committee or Institutional Review Board** | **Department / Organization** | **EC/IRB**  **City** | **EC/IRB**  **State/ Province** | **EC/IRB**  **Postal Code** | **Center Country** |
| --- | --- | --- | --- | --- | --- | --- |
| 3900 | Beacon Hospital Research Ethics Committee | Beacon Clinic | Sandyford |  | 18 | Ireland |
| 3901 | Research Ethics Committee Southeast Area | Whitfield Clinic | Waterford |  | X91 | Ireland |
| 3902 | Mater Misericordiae Univ Hospital Research Ethics Committee | Mater Private Hospital | Dublin | Dublin | 7 | Ireland |
| 3903 | Research Ethics Committee Southeast Area | Waterford Regional Hospital | Waterford |  | X91 | Ireland |
| 8400 | Helsinki Committee, Rabin MC | Rabin Medical Center-Beilinson Campus | Petah Tikva |  | 49100 | Israel |
| 8401 | Helsinki Committee, Sourasky MC | Tel Aviv Sourasky Medical Center | Tel-Aviv |  | 64239 | Israel |
| 8402 | Helsinki Committee, Bnai- Zion | Bnai Zion Medical Center | Haifa |  | 31048 | Israel |
| 6003 | Prasad Eye Institute Ethics Committee | L. V. Prasad Eye Institute | Hyderabad | Andhra Pradesh | 500034 | India |
| 6002 | All India Institute of Medical Sciences Ethics Committee | All India Institute of Medical Sciences | Ansari Nagar | Delhi | 110029 | India |
| 6000 | Aravind Eye Care System Ethical Committee | Aravind Eye Hospital | Coimbator e | Tamilnadu | 641014 | India |
| 6004 | Sangini Hospital Ethics Committee | Bankers Eye Institute | Ahmedaba d | Gujarat | 380006 | India |
| 6005 | Sparsh Hospital Ethics Committee | L. V. Prasad Eye Institute | Bhubanes war | Orissa | 751007 | India |
| 6008 | Vision Research Foundation Ethics Sub- Committee | Sankara Nethralaya | Chennai | Tamilnadu | 600006 | India |
| 6010 | National Institute of Ophthalmology Ethics Committee | National Institute of Ophthalmology | Pune | Maharasht ra | 411005 | India |
| 4001 | Comitato Etico Interaziendale | Reparto diOculistica dell Ospedale SS Antonio e Biagio e Cesare Arrigo de Alessandria | Alessandri a |  | 15121 | Italy |
| 4002 | Comitato Etico Indipendente - Azienda Ospedaliero-Univeritaria | Ente Ecclesiastico Ospedale Generale Regionale F Miulli | Bari | Bari | 70124 | Italy |
| 4003 | Reggio Calabria Comitato Etico | Azienda Ospedaliera Bianchi Melacrino Morelli | Reggio Calabria |  | 89100 | Italy |
| 4004 | Comitato Etico Dell 'Universita' Cattolica Del Sacro Cuore | Policlinico Universitario Agostino Gemelli | Roma |  | 001 68 | Italy |
| 4005 | Spedali Civili - Brescia Comitato Etico Provinciale | Azienda Socio Sanitaria Territoriale degli Spedali Civili di Brescia (Presidio Spedali Civili) | Brescia |  | 25123 | Italy |
| 4006 | Comitato per la Sperimentazione Clinica Medicinali dell'Az Ospedaliero Universitaria Pisana d. Pisa | Azienda Ospedaliero Universitaria Cisanello | Pisa |  | 56126 | Italy |
| 4008 | Regione Autonoma Della Sardegna Azienda Ospedaliero Universitaria Di Cagliari Comitato Etico Indipendente | Ospedale S.Giovanni di Dio | Cagliari |  | 091 24 | Italy |
| 4009 | Comitato Etico Dell 'Universita' Dell' Universita' "Sapienza" | Umberto I Pol. di Roma-Universita di Roma La Sapienza | Roma |  | 001 61 | Italy |
| 4010 | Regione del Veneto Aziendo U.L.S.S. n. 1 | Ospedale San Martino di Belluno | Belluno |  | 27717 | Italy |

| **Center Number** | **Ethics Committee or Institutional Review Board** | **Department / Organization** | **EC/IRB**  **City** | **EC/IRB**  **State/ Province** | **EC/IRB**  **Postal Code** | **Center Country** |
| --- | --- | --- | --- | --- | --- | --- |
| 6154 | Fukushima Medical University Hospital Institutional Review Board | Fukushima Medical University Hospital | Fukushima  -shi | Fukushim a-Ken | 960-1295 | Japan |
| 6155 | Sapporo City General Hospital The Ethical Committee of Sapporo City General Hospital | Sapporo City General Hospital | Sapporo- shi | Hokkaido | 060-8604 | Japan |
| 6115 | Asahikawa Medical University Hospital Institutional Review Board | Asahikawa Medical University Hospital | Asahikawa  -shi | Hokkaido | 078-8510 | Japan |
| 6161 | Tohoku University Hospital Institutional Review Board | Tohoku University Hospital | Sendai-shi | Miyagi- Ken | 980-8574 | Japan |
| 6116 | Akita University Hospital Institutional Review Board | Akita University Hospital | Akita-shi | Akita-Ken | 010-8543 | Japan |
| 6100 | Hattori Clinic Institutional Review Board | Shuhokai Ohtsuka Eye Hospital | Sapporo- shi | Hokkaido | 001-0016 | Japan |
| 6175 | Aomori Prefectural Central Hospital the ethical committee in Aomori Prefectural Central Hospital | Aomori Prefectural Central Hospital | Aomori | Aomori | 030-8553 | Japan |
| 6117 | Kitami Red Cross Hospital Institutional Review Board | Kitami Red Cross Hospital | Kitami-shi | Hokkaido | 090-8666 | Japan |
| 6118 | Jusendo General Hospital Independent Ethics Committee | Jusendo General Hospital | Koriyama- shi | Fukushim a-Ken | 963-8585 | Japan |
| 6182 | Yamagata University Hospital Institutional Review Board | Yamagata University Hospital | Yamagata- shi | Yamagata  -Ken | 990-9585 | Japan |
| 6101 | Hattori Clinic Institutional Review Board | Yoshida Eye Hospital | Hakodate- shi | Hokkaido | 041-0851 | Japan |
| 6119 | Gunma University Hospital Institutional Review Board | Gunma University Hospital | Maebashi- shi | Gunma- Ken | 371-8511 | Japan |
| 6107 | Tokyo Medical University Ibaraki Medical Center Institutional Review Board | Tokyo Medical University Ibaraki Medical Center | Inashiki- gun | Ibaraki- Ken | 300-0395 | Japan |
| 6102 | Hattori Clinic Institutional Review Board | Aoyagi Eye Clinic | Ueda-shi | Nagano- Ken | 386-0002 | Japan |
| 6120 | Dokkyo Medical University Hospital Institutional Review Board | Dokkyo Medical University Hospital | Shimotsug a-gun | Tochigi- Ken | 321-0293 | Japan |
| 6121 | Jichi Medical University Hospital Institutional Review Board | Jichi Medical University Hospital | Shimotsuk e-shi | Tochigi- Ken | 329-0498 | Japan |
| 6103 | Matsumoto Dental University Hospital Institutional Review Board | Matsumoto Dental University Hospital | Shiojiri-shi | Nagano- Ken | 399-0781 | Japan |
| 6122 | Tsukuba University Hospital Institutional Review Board | Tsukuba University Hospital | Tsukuba- shi | Ibaraki- Ken | 305-8576 | Japan |
| 6162 | Saitama Medical University Hospital Institutional Review Board | Saitama Medical University Hospital | Iruma-gun | Saitama- Ken | 350-0495 | Japan |
| 6163 | Toho University Sakura Medical Center Independent Ethics Committee | Toho University Sakura Medical Center | Sakura-shi | Chiba-Ken | 285-8741 | Japan |
| 6199 | Juntendo University Urayasu Hospital Institutional Review Board | Juntendo University Urayasu Hospital | Urayasu- shi | Chiba-Ken | 279-0021 | Japan |
| 6123 | Saitama Red Cross Hospital Institutional Review Board | Saitama Red Cross Hospital | Saitama- shi | Saitama- Ken | 338-8553 | Japan |
| 6156 | Hattori Clinic Institutional Review Board | Hoeikai Hoshiai Eye Clinic | Saitama- shi | Saitama- Ken | 336-0963 | Japan |
| 6104 | Yokohama City University Medical Center Institutional Review Board | Yokohama City University Medical Center | Yokohama  -shi | Kanagawa  -Ken | 232-0024 | Japan |

| **Center Number** | **Ethics Committee or Institutional Review Board** | **Department / Organization** | **EC/IRB**  **City** | **EC/IRB**  **State/ Province** | **EC/IRB**  **Postal Code** | **Center Country** |
| --- | --- | --- | --- | --- | --- | --- |
| 6124 | Yokohama City University Hospital Institutional Review Board | Yokohama City University Hospital | Yokohama  -shi | Kanagawa  -Ken | 236-0004 | Japan |
| 6125 | Yokosuka Kyosai Hospital Institutional Review Board | Yokosuka Kyosai Hospital | Yokohama  -shi | Kanagawa  -Ken | 236-0004 | Japan |
| 6183 | Seirei Hamamatsu General Hospital Institutional Review Board | Seirei Hamamatsu General Hospital | Hamamats u-shi | Shizuoka- Ken | 430-8558 | Japan |
| 6108 | St. Marianna University School of Medicine Hospital Institutional Review Board | St. Marianna University School of Medicine Hospital | Kawasaki- shi | Kanagawa  -Ken | 216-8511 | Japan |
| 6126 | Shizuoka Saiseikai General Hospital Internal Review Board | Shizuoka Saiseikai General Hospital | Shizuoka- shi | Shizuoka- Ken | 422-8527 | Japan |
| 6127 | Juntendo University Shizuoka Hospital Institutional Review Board | Juntendo University Shizuoka Hospital | Izunokuni- shi | Shizuoka- Ken | 410-2295 | Japan |
| 6109 | Hattori Clinic Institutional Review Board | Ishikawa Eye Clinic | Shizuoka- shi | Shizuoka- Ken | 420-0841 | Japan |
| 6176 | Hattori Clinic Institutional Review Board | Kaiya Ophthalmology | Hamamats u-shi | Shizuoka- Ken | 430-0903 | Japan |
| 6105 | Hattori Clinic Institutional Review Board | Totsukaekimae Suzuki Eye Clinic | Yokohama  -shi | Kanagawa  -Ken | 244-0003 | Japan |
| 6177 | Nihon University Hospital Institutional Review Board | Nihon University Hospital | Chiyoda- ku | Tokyo-To | 101-8309 | Japan |
| 6164 | Kyorin University Hospital Institutional Review Board | Kyorin University Hospital | Mitaka-shi | Tokyo-To | 181-8611 | Japan |
| 6165 | Tokyo Medical University Hospital Institutional Review Board | Tokyo Medical University Hospital | Shinjuku- ku | Tokyo-To | 160-0023 | Japan |
| 6128 | University of Yamanashi Hospital Institutional Review Board | University of Yamanashi Hospital | Chuo-shi | Yamanash i-Ken | 409-3898 | Japan |
| 6129 | Saiando Ochanomizu Inoue Eye Clinic Institutional Review Board | Saiando Ochanomizu Inoue Eye Clinic | Chiyoda- ku | Tokyo-To | 101-0062 | Japan |
| 6130 | Tokyo Medical University Hachioji Medical Center Institutional Review Board | Tokyo Medical University Hachioji Medical Center | Hachioji- shi | Tokyo-To | 193-0998 | Japan |
| 6187 | Juntendo University Hospital Institutional Review Board | Juntendo University Hospital | Bunkyo-ku | Tokyo-To | 113-8431 | Japan |
| 6173 | Tokyo Women's Medical University Hospital Institutional Review Board | Tokyo Women's Medical University Hospital | Shinjuku- ku | Tokyo-To | 162-8666 | Japan |
| 6131 | Nagoya University Hospital Institutional Review Board | Nagoya University Hospital | Nagoya- shi | Aichi-Ken | 466-8560 | Japan |
| 6132 | Mie University Hospital Internal Review Board | Mie University Hospital | Tsu-shi | Mie-Ken | 514-8507 | Japan |
| 6133 | Hattori Clinic Institutional Review Board | Shozankai Miyake Eye Hospital | Nagoya- shi | Aichi-Ken | 462-0825 | Japan |
| 6134 | JCHO Chukyo Hospital Institutional Review Board | JCHO Chukyo Hospital | Nagoya- shi | Aichi-Ken | 457-8510 | Japan |
| 6188 | Fujita Health University Hospital Institutional Review Board | Fujita Health University Hospital | Toyoake- shi | Aichi-Ken | 470-1192 | Japan |
| 6110 | Hattori Clinic Institutional Review Board | Koyokai Yayoi Hospital | Toyohashi  -shi | Aichi-Ken | 441-8106 | Japan |
| 6178 | Aichi Medical University Hospital Institutional Review Board | Aichi Medical University Hospital | Nagakute- shi | Aichi-Ken | 480-1195 | Japan |
| 6136 | Hattori Clinic Institutional Review Board | Nishijima Eye Clinic | Kyoto-shi | Kyoto-Fu | 604-0837 | Japan |
| 6157 | Shiga University of Medical Science Hospital Institutional Review Board | Shiga University of Medical Science Hospital | Otsu-shi | Shiga-Ken | 520-2192 | Japan |

| **Center Number** | **Ethics Committee or Institutional Review Board** | **Department / Organization** | **EC/IRB**  **City** | **EC/IRB**  **State/ Province** | **EC/IRB**  **Postal Code** | **Center Country** |
| --- | --- | --- | --- | --- | --- | --- |
| 6189 | Kyoto University Hospital Institutional Review Board | Kyoto University Hospital | Kyoto-shi | Kyoto-Fu | 606-8507 | Japan |
| 6158 | Kanazawa University Hospital Institutional Review Board | Kanazawa University Hospital | Kanazawa  -shi | Ishikawa- Ken | 920-8641 | Japan |
| 6190 | Toyama University Hospital Institutional Review Board | Toyama University Hospital | Toyama- shi | Toyama- Ken | 930-0194 | Japan |
| 6166 | Osaka University Hospital Institutional Review Board | Osaka University Hospital | Suita-shi | Osaka-Fu | 565-0871 | Japan |
| 6179 | Kansai Medical University Hospital Institutional Review Board | Kansai Medical University Hospital | Hirakata- shi | Osaka-Fu | 573-1191 | Japan |
| 6191 | Hyogo College of Medicine Hospital Institutional Review Board | Hyogo College of Medicine Hospital | Nishinomiy a-shi | Hyogo- Ken | 663-8501 | Japan |
| 6200 | Hyogo Prefectural Amagasaki General Medical Center Institutional Review Board | Hyogo Prefectural Amagasaki General Medical Center | Amagasak i-shi | Hyogo- Ken | 660-8550 | Japan |
| 6137 | Kansai Medical University Medical Center Institutional Review Board | Kansai Medical University Takii Hospital | Moriguchi- shi | Osaka-Fu | 570-8507 | Japan |
| 6138 | Steel Memorial Hirohata Hospital Institutional Review Board | Steel Memorial Hirohata Hospital | Himeji-shi | Hyogo- Ken | 671-1122 | Japan |
| 6159 | Nara Medical University Hospital Institutional Review Board | Nara Medical University Hospital | Kashihara- shi | Nara-Ken | 634-8522 | Japan |
| 6184 | Kobe Kaisei Hospital Institutional Review Board | Kobe Kaisei Hospital | Kobe-shi | Hyogo- Ken | 657-0068 | Japan |
| 6160 | Kindai University Hospital Independent Ethics Committee | Kindai University Hospital | Osakasay ama-shi | Osaka-Fu | 589-8511 | Japan |
| 6202 | Japan Red Cross Society Wakayama Medical Center Independent Ethics Committee | Japan Red Cross Society Wakayama Medical Center | Wakayam a-shi | Wakayam a-Ken | 640-8558 | Japan |
| 6180 | Kakogawa City West Hospital Institutional Review Board | Kakogawa City West Hospital | Kakogawa  -shi | Hyogo- Ken | 675-8611 | Japan |
| 6139 | Kagawa University Hospital Institutional Review Board | Kagawa University Hospital | Kita-gun | Kagawa- Ken | 761-0793 | Japan |
| 6140 | Hiroshima University Hospital Institutional Review Board | Hiroshima University Hospital | Hiroshima- shi | Hiroshima- Ken | 734-8551 | Japan |
| 6141 | Ehime University Hospital Independent Ethics Committee | Ehime University Hospital | Toon-shi | Ehime- Ken | 791-0295 | Japan |
| 6185 | Tokushima University Hospital Institutional Review Board | Tokushima University Hospital | Tokushima  -shi | Tokushim a-Ken | 770-8503 | Japan |
| 6167 | Kochi Medical School Hospital Institutional Review Board | Kochi Medical School Hospital | Nankoku- shi | Kochi-Ken | 783-8505 | Japan |
| 6111 | Matsuyama Red Cross Hospital Institutional Review Board | Matsuyama Red Cross Hospital | Matsuyam a-shi | Ehime- Ken | 790-8524 | Japan |
| 6192 | Kyushu University Hospital Institutional Review Board | Kyushu University Hospital | Higashi-ku | Fukuoka | 812-8582 | Japan |
| 6143 | Japanese Red Cross Nagasaki Genbaku Hospital Independent Ethics Committee | Japanese Red Cross Nagasaki Genbaku Hospital | Nagasaki- shi | Nagasaki- Ken | 852-8511 | Japan |
| 6144 | Kagoshima University Hospital Institutional Review Board | Kagoshima University Medical And Dental Hospital | Kagoshim a-shi | Kagoshim a-Ken | 890-8520 | Japan |
| 6193 | University of Miyazaki Hospital Institutional Review Board | University of Miyazaki Hospital | Miyazaki- shi | Miyazaki- Ken | 889-1692 | Japan |

| **Center Number** | **Ethics Committee or Institutional Review Board** | **Department / Organization** | **EC/IRB**  **City** | **EC/IRB**  **State/ Province** | **EC/IRB**  **Postal Code** | **Center Country** |
| --- | --- | --- | --- | --- | --- | --- |
| 6168 | Meiwakai Miyata Ophthalmic Hospital Institutional Review Board | Meiwakai Miyata Ophthalmic Hospital | Miyakonoj o-shi | Miyazaki- Ken | 885-0051 | Japan |
| 6194 | Fukuoka University Hospital Institutional Review Board | Fukuoka University Hospital | Fukuoka- shi | Fukuoka- Ken | 814-0180 | Japan |
| 6145 | Nagasaki University Hospital Institutional Review Board | Nagasaki University Hospital | Nagasaki- shi | Nagasaki- Ken | 852-8501 | Japan |
| 6195 | University of Occupational and Environmental Health Hospital Internal Review Board | University of Occupational and Environmental Health Hospital | Kitakyushu  -shi | Fukuoka- Ken | 807-8556 | Japan |
| 6169 | Meiwakai Kagoshima Miyata Ophthalmic Hospital Institutional Review Board | Meiwakai Kagoshima Miyata Ophthalmic Hospital | Kagoshim a-shi | Kagoshim a-Ken | 890-0046 | Japan |
| 6106 | Hattori Clinic Institutional Review Board | Taidokai Sato Ganka Iin Domachi Clinic | Yamagata- shi | Yamagata  -Ken | 990-0051 | Japan |
| 6146 | Yonezawa City Hospital Institutional Review Board | Yonezawa City Hospital | Yonezawa  -shi | Yamagata  -Ken | 992-8502 | Japan |
| 6170 | JOHAS Tohoku Rosai Hospital Institutional Review Board | JOHAS Tohoku Rosai Hospital | Sendai-shi | Miyagi- Ken | 981-8563 | Japan |
| 6114 | Meiji University of Integrative Medicine Institutional Review Board | Meiji University of Integrative Medicine | Nantan-shi | Kyoto-Fu | 629-0392 | Japan |
| 6174 | Obihiro Kyokai Hospital Institutional Review Board | Obihiro Kyokai Hospital | Obihiro-shi | Hokkaido | 080-0805 | Japan |
| 6171 | Sapporo Medical University Hospital Institutional Review Board | Sapporo Medical University Hospital | Sapporo- shi | Hokkaido | 060-8543 | Japan |
| 6148 | Ogaki Tokushukai Hospital Tokushu-kai Ethics Committee | Ogaki Tokushukai Hospital | Ogaki-shi | Gifu-Ken | 503-0015 | Japan |
| 6112 | Hattori Clinic Institutional Review Board | Tagawa Eye Clinic | Kanazawa  -shi | Ishikawa- Ken | 920-1151 | Japan |
| 6186 | University of Fukui Hospital Institutional Review Board | University of Fukui Hospital | Yoshida- gun | Fukui-Ken | 910-1193 | Japan |
| 6150 | Japanese Red Cross Society Suwa Hospital Institutional Review Board | Japanese Red Cross Society Suwa Hospital | Suwa-shi | Nagano- Ken | 392-8510 | Japan |
| 6151 | Hattori Clinic Institutional Review Board | Hirota Eye Clinic | Shunan- shi | Yamaguch i-Ken | 745-0017 | Japan |
| 6113 | Hattori Clinic Institutional Review Board | Dannoue Eye Clinic | Kawasaki- shi | Kanagawa  -Ken | 211-0053 | Japan |
| 6152 | Hattori Clinic Institutional Review Board | Jigankai Sanjo Eye Clinic | Sanjo-shi | Niigata- Ken | 955-0852 | Japan |
| 6153 | Hattori Clinic Institutional Review Board | Infinity Medical Group Kondo Eye Clinic | Hachioji- shi | Tokyo-To | 192-0081 | Japan |
| 6172 | Hattori Clinic Institutional Review Board | Musashi Dream Eye Clinic | Osaka-shi | Osaka-Fu | 543-0027 | Japan |
| 6181 | Toho University Ohashi Medical Center Institutional Review Board | Toho University Ohashi Medical Center | Meguro-ku | Tokyo-To | 153-8515 | Japan |
| 6196 | Hattori Clinic Institutional Review Board | Shinseikai Toyama Hospital | Imizu-shi | Toyama- Ken | 939-0243 | Japan |
| 6197 | Hattori Clinic Institutional Review Board | Ando Eye Clinic | Ashigarak ami-gun | Kanagawa  -Ken | 258-0003 | Japan |
| 6198 | Juntendo University Nerima Hospital Institutional Review Board | Juntendo University Nerima Hospital | Nerima-ku | Tokyo-To | 177-8521 | Japan |
| 6600 | Kangnam Sacred Heart Hospital IRB Membership List | Hallym University Kangnam Sacred Heart Hospital | Seoul |  | 150-950 | Korea, Republic of |
| 6601 | Seoul National University Bundang Hospital | Seoul National University Bundang Hospital | Seongnam  -si | Gyeonggi- do | 13620 | Korea, Republic of |

| **Center Number** | **Ethics Committee or Institutional Review Board** | **Department / Organization** | **EC/IRB**  **City** | **EC/IRB**  **State/ Province** | **EC/IRB**  **Postal Code** | **Center Country** |
| --- | --- | --- | --- | --- | --- | --- |
| 6602 | Seoul National University Hospital Institutional Review Board | Seoul National University Hospital | Seoul | Gyeonggi- do | 110744 | Korea, Republic of |
| 6603 | Kim's Eye Hospital IRB | Kim's Eye Hospital | Seoul |  | 150-034 | Korea, Republic of |
| 6604 | Seoul St. Mary's Hospital, The Catholic University of Korea IRB | The Catholic University of Korea, Seoul St. Mary’s Hospital | Seocho-gu |  | 137-701 | Korea, Republic of |
| 6605 | The Institutional Review Board of Kyungpook National University Hospital | Kyungpook National University Hospital | Daegu | Gyeongsa ngbuk-do | 700-721 | Korea, Republic of |
| 6606 | Yonsei University Gangnam Severance Hospital, Institutional Review Board | Gangnam Severance Hospital, Yonsei University Health System | Gangnam- gu |  | 062 73 | Korea, Republic of |
| 6607 | PNUH Institutional Review Board | Pusan National University Hospital | Busan |  | 602-739 | Korea, Republic of |
| 6608 | IRB of Inje University Busan Paik Hospital | Inje University Busan Paik Hospital | Busan |  | 47392 | Korea, Republic of |
| 6609 | Yeungnam University Hospital Institutional Review Board | Yeungnam University Hospital | Daegu |  | 42415 | Korea, Republic of |
| 6610 | Asan Medical Center IRB | Asan Medical Center | Seoul |  | 055 05 | Korea, Republic of |
| 6612 | KHUH IRB | Kyung Hee University Hospital | Seoul |  | 024 47 | Korea, Republic of |
| 6652 | Samsung Medical Center Institutional Review Board | Samsung Medical Center | Seoul |  | 063 51 | Korea, Republic of |
| 6611 | The Institutional Review Board of Ajou University Hospital | Ajou University Hospital | Gyeonggi- do | Gyeonggi- do | 16499 | Korea, Republic of |
| 1400 | Comite de Etica en Investigacion de la facultad de Medicina y Hospital Universitario de la Universidad Autonoma de Nuevo Leon | Hospital Universitario Dr Jose E Gonzalez | Monterrey | Nuevo León | 64460 | Mexico |
| 1401 | Comite de Etica en Investigacion de la Escuela de Medicina del Instituto Tecnologico y de Estudios Superiores de Monterrey | CIIES | Monterrey | Nuevo León | 64710 | Mexico |
| 1403 | Comite de Investigacion Instituo de Oftalmologia Fundacion de Asistencia Privada | Instituto de Oftalmología Fundación Conde de la Valenciana | Mexico City | Distrito Federal | 0 6800 | Mexico |
| 1405 | Comite de Etica e Investigacion Fundacion Hospital "Nuestra Senora de la Luz" | Hospital Oftalmológico Nuestra Señora de la Luz | Mexico City | Distrito Federal | 0 6030 | Mexico |
| 1406 | Comite de Investigaction de la Clinica Bajio CLINBA S.C. | Clínica de Ojos Monterrey S.A. de C.V. | Guanajuat o | Nuevo León | 36090 | Mexico |
| 1412 | Comite Independiente de Etica de Investigación y Bioseguridad del Bajio SC | Dr. Alejandro Dalma y asoc. | Guanajuat o | Distrito Federal | 36090 | Mexico |
| 1413 | Comite Independiente de Etica de Investigación y Bioseguridad del Bajio SC | RetimediQ Centro de Retina y Oftalmologia Especializada | Guanajuat o | Yucatán | 36090 | Mexico |
| 5100 | Medical Ethics Committee, University Malaya Medical Centre | University of Malaya Eye Research Centre | Kuala Lumpur | Kuala Lumpur | 59100 | Malaysia |
| 5101 | Medical Research Ethics Committee, Ministry of Health Malaysia | Hospital Selayang | Kuala Lumpur | Selangor | 59000 | Malaysia |

| **Center Number** | **Ethics Committee or Institutional Review Board** | **Department / Organization** | **EC/IRB**  **City** | **EC/IRB**  **State/ Province** | **EC/IRB**  **Postal Code** | **Center Country** |
| --- | --- | --- | --- | --- | --- | --- |
| 5102 | Medical Research & Ethics Committee Kementerian Kesihatan Malaysia | International Specialist Eye Centre | Kuala Lumpur | Kuala Lumpur | 59000 | Malaysia |
| 5103 | Universiti Kebangsaan Malaysia Medical Centre | Pusat Perubatan Universiti Kebangsaan Malaysia | Kuala Lumpur | Kuala Lumpur | 56000 | Malaysia |
| 4100 | Medisch Ethische Toetsingscommissie | Sint Elisabeth Ziekenhuis Afd. Oogheelkunde | Tilburg | Noord- Brabant | 5022 GC | Netherlands |
| 4101 | Medisch Ethische Toetsingscommissie | OMC Amsterdam | Tilburg | Noord- Brabant | 5022 GC | Netherlands |
| 4103 | Medisch Ethische Toetsingscommissie | Flevoziekenhuis | Tilburg | Noord- Brabant | 5022 GC | Netherlands |
| 8500 | Hospital Nacional Guillermo Almenara Irigoyen Comite de Etica en Investigacion | Hospital Nacional Guillermo Almenara Irigoyen | Lima |  | 15036 | Peru |
| 8501 | Comite Institucional de Etica en Investigacion de la Universidad de San Martin de Porres | Instituto Oftalmosalud S.R.L | Lima |  | 15036 | Peru |
| 8502 | Comite Institucional de Etica en Investigacion de la Universidad de San Martin de Porres | Macula D&T | Lima |  | 15036 | Peru |
| 8503 | Prisma ONG | Ophtalmology-TG Laser Oftalmica | Lima |  | 15036 | Peru |
| 4200 | Ethics Committee of Silesian Medical Chamber | Samodzielny Publiczny ZOZ | Grazynski ego |  | 40-126 | Poland |
| 4205 | Ethics Committee of Silesian Medical Chamber | NZOZ Lens-Med | Grazynski ego |  | 40-126 | Poland |
| 4201 | Ethics Committee of Silesian Medical Chamber | 10 Wojskowy Szpital Kliniczny | Grazynski ego |  | 40-126 | Poland |
| 4206 | Ethics Committee of Silesian Medical Chamber | Szpital Specjalistyczny im Sokołowskiego | Grazynski ego |  | 40-126 | Poland |
| 4202 | Ethics Committee of Silesian Medical Chamber | NZOZ Ocu Service | Grazynski ego |  | 40-126 | Poland |
| 4203 | Ethics Committee of Silesian Medical Chamber | Specjalistyczny Cabinet Lekarski Krystyna Raczynska | Grazynski ego |  | 40-126 | Poland |
| 4204 | Ethics Committee of Silesian Medical Chamber | NZOZ Medilens | Grazynski ego |  | 40-126 | Poland |
| 4211 | Ethics Committee of Silesian Medical Chamber | Wojewódzki Szpital Okulistyczny w Krakowie | Grazynski ego |  | 40-126 | Poland |
| 4212 | Ethics Committee of Silesian Medical Chamber | Centrum Diagnostyki i Mikrochirurgii Oka LENS | Grazynski ego |  | 40-126 | Poland |
| 4213 | Ethics Committee of Silesian Medical Chamber | Szpital Specjalistyczny IM  J.K. Lukowicza | Grazynski ego |  | 40-126 | Poland |
| 4217 | Ethics Committee of Silesian Medical Chamber | Centrum Medyczne Uno-Med (Private Practice) | Grazynski ego |  | 40-126 | Poland |
| 4219 | Ethics Committee of Silesian Medical Chamber | Szpital Zakonu Bonifratrów im. Św. Jana Bożego w Łodzi | Grazynski ego |  | 40-126 | Poland |
| 4220 | Ethics Committee of Silesian Medical Chamber | Wojskowy Instytut Medyczny | Grazynski ego |  | 40-126 | Poland |
| 4221 | Ethics Committee of Silesian Medical Chamber | Medical University of Lublin | Grazynski ego |  | 40-126 | Poland |
| 4300 | Aibili Comissao de Etica para a Saude | AIBILI | Porto Salvo |  | 2740-262 | Portugal |
| 4303 | Hospital Lusiadas Lisboa Comissao de Etica para a  Saude | HPP - Hospital dos Lusíadas | Lisbon |  | 1500-458 | Portugal |

| **Center Number** | **Ethics Committee or Institutional Review Board** | **Department / Organization** | **EC/IRB**  **City** | **EC/IRB**  **State/ Province** | **EC/IRB**  **Postal Code** | **Center Country** |
| --- | --- | --- | --- | --- | --- | --- |
| 4304 | Centro Hospitalar de Entre o Douro e Vouga, E.P.E. | Centro Hospitalar de Entre o Douro e Vouga, E.P.E - Hospital de São Sebastião | Santa Maria da Feira |  | 4520-211 | Portugal |
| 4306 | Centro Hospitalar Leiria Comissao de Etica | Centro Hospitalar Leiria - Hospital Santo André | Leira |  | 2410-197 | Portugal |
| 4301 | Centro Hospitalar Leiria Comissao de Etica | Hospital Pedro Hispano | Leira |  | 2410-197 | Portugal |
| 4302 | Centro Hospitalar Do Baixo Vouga, E.P.E./Aveiro | Centro Hospitalar do Baixo Vouga, E.P.E.  – Unidade de Aveiro | Aveiro |  | 3814-501 | Portugal |
| 4305 | CEIC - Parque de Saude de Lisboa | Espaço Médico de Coimbra | Lisboa |  | 1749-004 | Portugal |
| 4407 | The Committee of Biomedical Ethics of Ufa Institute of scientific- research of eye diseases of Academy of Sciences (Bashkortostan) | SBI "Ufa scientific research institute of eye diseases of academy of sciences of the republic of Bas | Ufa |  | 450077 | Russia |
| 4400 | The Ethics Committee of "The Postgraduating Doctors' Training Institute" of the Healthcare and Social Development Ministry of the Chuvash Republic | The S.N.Fyodorov Federal State Institution Eye Microsurgery Complex (Cheboxary) | Cheboksar y |  | 428003 | Russia |
| 4412 | The Ethics Committee of GBUZ SOCOB n.a. T.I.  Eroshevsky | SBEI HPE "Samara State Medical University" of the MoH of the RF |  |  | 443099 | Russia |
| 4423 | The Independent Multidisciplinary Committee on Ethical Review of Clinical Trials | The Irkutsk Affiliate of Federal State Budgetary Institution "MNTK ye  Microsurgery Complex" n.a. S.N | Moscow |  | 125468 | Russia |
| 4403 | The Independent Multidisciplinary Committee on Ethical Review of Clinical Trials | Institution of Republic Sakha (Yakutiya) Yakutsk Republican Ophthalmology Hospital | Moscow |  | 125468 | Russia |
| 4402 | The Independent Multidisciplinary Committee on Ethical Review of Clinical Trials | The S.N.Fyodorov Federal State Institution Eye Microsurgery Complex (Khabarovsk) | Moscow |  | 125468 | Russia |
| 4404 | The Independent Multidisciplinary Committee on Ethical Review of Clinical Trials | Territorial Diabetic Center | Moscow |  | 125468 | Russia |
| 4401 | The Independent Multidisciplinary Committee on Ethical Review of Clinical Trials | Dignostic Center №7 | Moscow |  | 125468 | Russia |
| 4406 | The Ethics Committee of "Federal State budgetary Institution "Scientific Research Institute of Eye Diseases" of Russian Academy of medical Sciences | Scientific Research Institute of Eye Diseases | Moscow |  | 119021 | Russia |
| 4408 | The Ethics Committee of The Helmholtz Moscow Research Institute of Eye Diseases | Moscow Helmholtz Research Institute of Ophthalmology | Moscow |  | 105062 | Russia |

| **Center Number** | **Ethics Committee or Institutional Review Board** | **Department / Organization** | **EC/IRB**  **City** | **EC/IRB**  **State/ Province** | **EC/IRB**  **Postal Code** | **Center Country** |
| --- | --- | --- | --- | --- | --- | --- |
| 4405 | The Independent Multidisciplinary Committee on Ethical Review of Clinical Trials | Tyumen Regional Ophthalmology Dispensary | Moscow |  | 125468 | Russia |
| 4419 | The Independent Multidisciplinary Committee on Ethical Review of Clinical Trials | Chita State Medical Academy | Moscow |  | 125468 | Russia |
| 4418 | The Independent Multidisciplinary Committee on Ethical Review of Clinical Trials | The S.N.Fyodorov Federal State Institution Eye Microsurgery Complex (Tambov) | Moscow |  | 125468 | Russia |
| 4420 | The Independent Multidisciplinary Committee on Ethical Review of Clinical Trials | SBHI "Penza Regional Ophtalmological Hospital" | Moscow |  | 125468 | Russia |
| 4421 | The Independent Multidisciplinary Committee on Ethical Review of Clinical Trials | BI of Khanty- Mansyisk region Yugra "Surgut regional clinical hospital" | Moscow |  | 125468 | Russia |
| 4422 | The Independent Multidisciplinary Committee on Ethical Review of Clinical Trials | Krasnodar Branch of The S.N. Fyodorov FSBI "Eye  microsurgery complex" | Moscow |  | 125468 | Russia |
| 4425 | The Independent Multidisciplinary Committee on Ethical Review of Clinical Trials | SBEI HPE "Saratov State Medical University n.a. V. I. Razumovskiy" of the MoH of the RF | Moscow |  | 125468 | Russia |
| 4426 | State Autonomous Healthcare Institution "Republican Clinical Opthalmologic Hospital of the Ministry of Health of Tatarstan Republic" | SAIH "Republican clinical ophthalmological hospital of MoH of Rebublic of Tatarstan" | Kazan |  | 420012 | Russia |
| 4424 | Biomedical Ethics Committee of Federal State Budgetary Institution of scientific and technical complex of Microsurgical eye | MBHI City Clinical Hospital #11 | Novosibirs k |  | 630071 | Russia |
| 4427 | Biomedical Ethics Committee of Federal State Budgetary Institution of scientific and technical complex of Microsurgical eye | The S.N.Fyodorov Federal State Institution Eye Microsurgery Complex (Novosibirsk) | Novosibirs k |  | 630071 | Russia |
| 8901 | Prince Sultan Military Medical City Research Ethics Committee | Prince Sultan Military Medical City | Riyadh |  | 11159 | Saudi Arabia |
| 8902 | Kingdom of Saudi Arabia  - Ministry of National Guard - Health Affairs | King Abdullah International Medical Research Center IRB Office | Riyadh |  | 22490 | Saudi Arabia |
| 8900 | King Khaled Eye Specialist Hospital HEC/IRB | King Khaled Eyes Specialist Hospital | Riyadh |  | 11462 | Saudi Arabia |
| 8903 | Kingdom of Saudi Arabia  - Ministry of National Guard - Health Affairs | King Abdullah International Medical Research Center IRB Office | Riyadh |  | 22490 | Saudi Arabia |
| 6300 | Parkway Hospitals Singapore Pte Ltd | PIEC | Singapore |  | 238164 | Singapore |
| 6301 | National Healthcare Group | NUHS | Singapore |  | 149547 | Singapore |
| 6302 | SingHealth Centralised Institutional Review Board | Singapore National Eye Centre | Singapore |  | 168753 | Singapore |
| 8600 | Republic of Slovenia The National Medical Ethics Committee | Univerzitetni Klinicni Center Ljubljana Ocesna Klinika | Ljubljana |  | SI-1525 | Slovenia |

| **Center Number** | **Ethics Committee or Institutional Review Board** | **Department / Organization** | **EC/IRB**  **City** | **EC/IRB**  **State/ Province** | **EC/IRB**  **Postal Code** | **Center Country** |
| --- | --- | --- | --- | --- | --- | --- |
| 8601 | Republic of Slovenia The National Medical Ethics Committee | University Medical Centre Maribor | Ljubljana |  | SI-1525 | Slovenia |
| 8602 | Ethical Committee General Hospital Celje | General Hospital Celje | Celje |  | 3000 | Slovenia |
| 8603 | Republic of Slovenia The National Medical Ethics Committee | General Hospital Novo Mesto | Ljubljana |  | SI-1525 | Slovenia |
| 4500 | *Nemocnica Ruzinov* | Univerzitna nemocnica Bratislava, Nemocnica Ruzinov | Bratislava |  | 82606 | Slovakia |
| 4502 | EK-Fakultna nemocnica Trencin | Fakultna nemocnica Trencin | Trencin |  | 91101 | Slovakia |
| 4504 | EK-Ustredna vojenska nemocnica SNP Ruzomberok | Ustredna vojenska nemocnica SNP Ruzomberok- Fakultna nemocnica | Ruzomber ok |  | 034 26 | Slovakia |
| 4507 | EK-Fakultna nemocnica s poliklinikou F.D. Roosevelta | NsP Banská Bystrica | Banska Bystrica |  | 975 17 | Slovakia |
| 4503 | EK-Fakultna nemocnica Zilina | Fakultna nemocnica s poliklinikou Zilina | Zilina |  | 012 07 | Slovakia |
| 4501 | Eticka komisia NsP Trebisov a.s. | Nemocnica s poliklinikou Trebisov a.s. | Trebisov |  | 075 01 | Slovakia |
| 4510 | EK-UN Bratislava, Nemocnica sv. Cyrila a Metoda | Univerzitna nemocnica Bratislava, Nemocnica sv. Cyrila a Metoda | Bratislava |  | 85107 | Slovakia |
| 4512 | EK-Nemocnica Poprad a.s. | Nemocnica Poprad a.s. | Poprad |  | 058 45 | Slovakia |
| 4513 | EK-FNsP Nove Zamky | Fakultna nemocnica s poliklinikou Nove Zamky | Nove Zamky |  | 94002 | Slovakia |
| 4514 | Oftal s.r.o, Specializovana nemocnica v odbore oftalmologia | Oftal s.r.o. | Zvolen |  | 960 01 | Slovakia |
| 4517 | Rozhodnutie Etickej komisie Nemocnica Svateho Michala, a.s., | Nemocnica svateho Michala | Bratislava |  | 811 08 | Slovakia |
| 4900 | Ankara University Ethics Committee | Hacettepe University Medical Faculty | Ankara |  | 061 00 | Turkey |
| 4901 | Ankara University Ethics Committee | Ankara University Medical Faculty | Ankara |  | 061 00 | Turkey |
| 4902 | Ankara University Ethics Committee | Bilim University Florence Nightingale Hospital | Ankara |  | 061 00 | Turkey |
| 4904 | Ankara University Ethics Committee | Gazi University Hospital | Ankara |  | 061 00 | Turkey |
| 4903 | Ankara University Ethics Committee | Ankara Ataturk Training and Research Hospital | Ankara |  | 061 00 | Turkey |
| 8700 | State Institution "The Filatov Institute of Eye Diseases and Tissue Therapy of the National Academy of Medical Sciences of Ukraine" | V.P.Filatov Institute of Eye Diseases and Tissue Therapy AMS | Odessa |  | 65061 | Ukraine |
| 8701 | Local Ethics Committee at the Oppthalmology Clinic Eye Microsurgery Center | City clinical ophthalmological hospital | Kyiv |  | 3680 | Ukraine |
| 8702 | Local Ethics Committee at the Communal Institution Dnipropetrovsk Regional Clinical Ophthalmological Hospital | Regional clinical hospital n.a.  Mechnikova | Dnipropetr ovsk |  | 49005 | Ukraine |
| 8703 | Local Ethics Committee at the Hospital | LLC “Lugansk Regional Central Eye Hospital” | Lugansk |  | 91055 | Ukraine |

| **Center Number** | **Ethics Committee or Institutional Review Board** | **Department / Organization** | **EC/IRB**  **City** | **EC/IRB**  **State/ Province** | **EC/IRB**  **Postal Code** | **Center Country** |
| --- | --- | --- | --- | --- | --- | --- |
| 8704 | Local Ethics Committee at the Kharkov Regional Clinical Hospital | Kharkov Regional Clinical Hospital | Kharkiv |  | 61022 | Ukraine |
| 9000 | Comité de Etica Independiente de la Fundación Dominicana de Infectología | Consultorio Oftalmológico Medicalnet | Santo Domingo | Santo Domingo | N/A | Dominican Republic |
| 9001 | Comité de Etica del Centro Cardio-Neuro Oftalmológico y Trasplante CECANOT | Centro Cardio-Neuro Oftalmológico y Trasplante (CECANOT) | Santo Domingo | Santo Domingo | N/A | Dominican Republic |
| 9031 | Instituto Conmemorativo Gorgas de Estudios de la Salud | Clínica de Vitreo y Macula Dra. Ana Paz | Panamá | Panamá | N/A | Panamá |
| 9033 | Instituto Conmemorativo Gorgas de Estudios de la Salud | Clinica Yee | Panamá | Panamá | N/A | Panamá |
| 9051 | Comité de Etica Zugueme | Clínica Oftalmológica Santa Clara | Guatemala | Guatemal a | 1015 | Guatemala |
| 9061 | Research Ethics Committee - Central Directorate for Research and Health Development | Ministry of Health and Population | Cairo | Cairo | 11516 | Egypt |
| 9062 | Research Ethics Committee - Central Directorate for Research and Health Development | Ministry of Health and Population | Cairo | Cairo | 11516 | Egypt |
| 9064 | Research Ethics Committee - Central Directorate for Research and Health Development | Ministry of Health and Population | Cairo | Cairo | 11516 | Egypt |
| 9065 | Research Ethics Committee - Central Directorate for Research and Health Development | Ministry of Health and Population | Cairo | Alexandria | 11516 | Egypt |
| 9066 | Research Ethics Committee - Central Directorate for Research and Health Development | Ministry of Health and Population | Cairo | Cairo | 11516 | Egypt |
| 9071 | Instituto Costarricense de Investigaciones Clínicas (ICIC) | Instituto de Cirugia Ocular | San José | San José | N/A | Costa Rica |
| 9072 | Instituto Costarricense de Investigaciones Clínicas (ICIC) | Clínica 20/20 | San José | San José | N/A | Costa Rica |
| 9073 | Instituto Costarricense de Investigaciones Clínicas (ICIC) | Oftalmocima | San José | San José | N/A | Costa Rica |
| 1500 | Centro Nacional de Bioetica | Unidad Oftalmologica de Caracas, C.A. Santa Paula | Caracas |  | 1060 | Venezuela |
| 1501 | Centro Nacional de Bioetica | Clinica de Especialidades Oftalmologicas (Retina & Vitreo) | Caracas |  | 1060 | Venezuela |
| 1502 | Centro Nacional de Bioetica | Centro Oftalmologico de Valencia (CEOVAL) | Caracas |  | 1060 | Venezuela |
| 1503 | Centro Nacional de Bioetica | Instituto Oftalmológico IUMO trinidad | Caracas |  | 1060 | Venezuela |

**Supplementary Figure 1. Country-wise baseline characteristics of treatment-naïve patients with CRVO**

**A. age (years), B. mean baseline VA, and C. median (days) time from diagnosis to first ranibizumab treatment.**


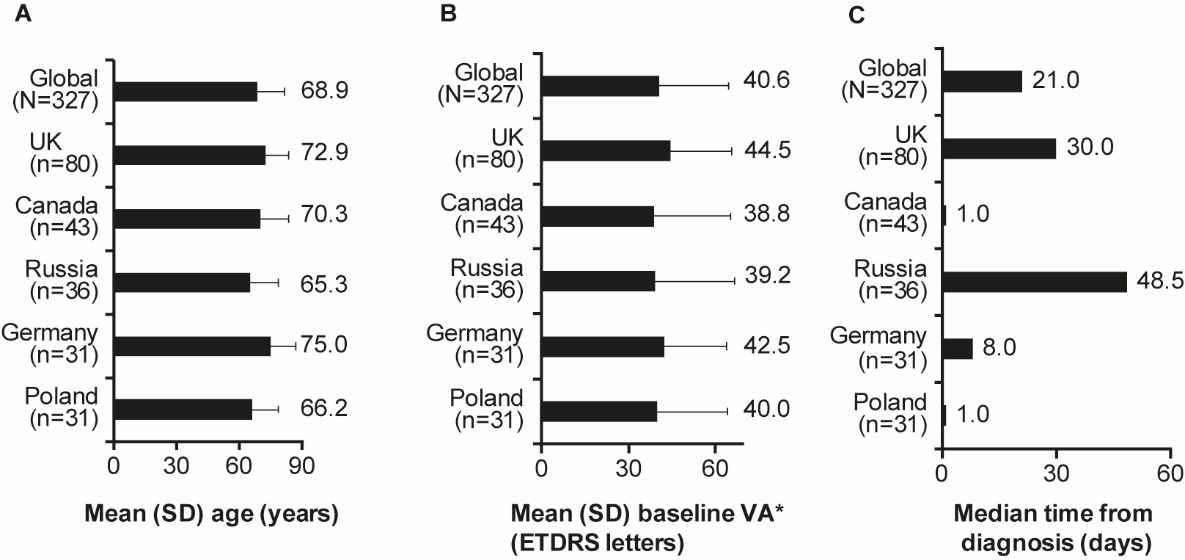


Countries that recruited >20 treatment-naïve CRVO patients.

*VA for patients with available data at baseline is shown.

CRVO, central retinal vein occlusion; ETDRS, Early Treatment Diabetic Retinopathy Study; VA, visual acuity

**Supplementary Figure 2. Mean change in VA from baseline at Year 1 in patients who received the loading dose and those who did not (Year-1 primary treated eye set).**


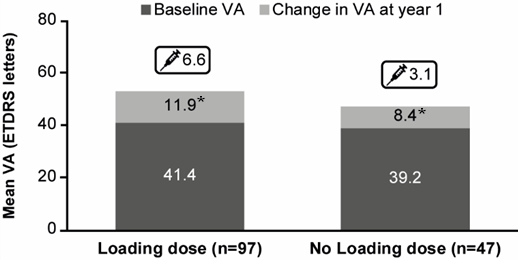


The Year-1 primary treated eye set included patients from the primary treated eye set who had both baseline and Year-1 data and had remained in the study for at least 365 days.

*95% CI (─3.14, 10.14) for the difference in VA gains at Year-1 in patients receiving and not receiving loading dose.

CI, confidence interval; ETDRS, Early Treatment Diabetic Retinopathy Study; n, number of patients; VA, visual acuity.

**Supplementary Figure 3. Proportion of patients with categorical loss or gain in VA at Year 1 (Year-1 primary treated eye set).**


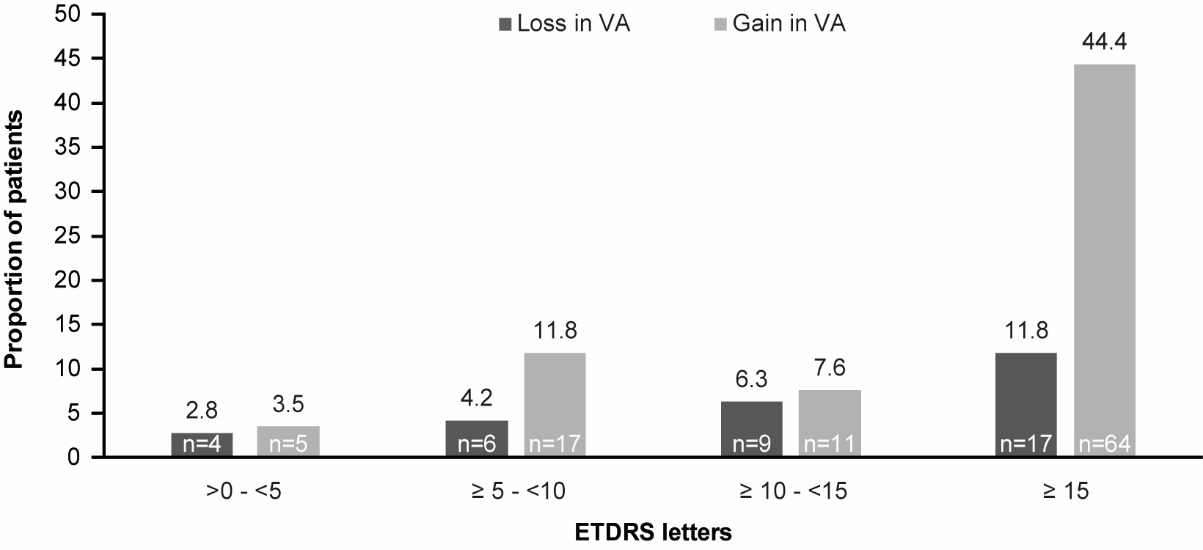


The Year-1 primary treated eye set included patients from the primary treated eye set who had both baseline and Year-1 data and had remained in the study for at least 365 days.

ETDRS, Early Treatment Diabetic Retinopathy Study; n, number of patients; VA, visual acuity.

**Supplementary Table 2. Ocular (study eye) AEs suspected to be related to ranibizumab treatment and/or ocular injection over 5 years (safety set).**

| **Preferred term, n (%)** | **Treatment-naïve patients with CRVO**  **n=327** |
| --- | --- |
| **Ocular AEs, total** | 11 (3.4) |
| Ocular hypertension | 4 (1.2) |
| Conjunctival hemorrhage | 3 (0.9) |
| Eye pain | 2 (0.6) |
| Amaurosis fugax | 1 (0.3) |
| Ocular hyperemia | 1 (0.3) |
| Photopsia | 1 (0.3) |
| Visual field defect | 1 (0.3) |

Indication and pre-treatment status refers to the primary treated eye.

Only AEs occurring during the safety observation period are included.

Preferred terms are presented within primary system organ class by descending order of frequency of total column.

A patient with multiple occurrences of an AE is counted only once in the AE category for that group.

Patients with a baseline visit date present are included.

Data collected until the last recorded follow-up date was used to perform the analyses.

AE, adverse event; CRVO; central retinal vein occlusion; n, number of patients

**Supplementary Table 3.** **Incidence of ocular and non-ocular serious adverse events over 5 years (safety set).**

| **Preferred term, n (%)** | **Treatment-naïve patients with CRVO**  **N=327** |
| --- | --- |
| **Ocular SAEs, total** | **4 (1.2)** |
| Glaucoma | 2 (0.6) |
| Amaurosis fugax | 1 (0.3) |
| Blindness | 1 (0.3) |
| Cataract traumatic | 1 (0.3) |
| **Non-ocular SAEs, total** | **22 (6.7)** |
| Pneumonia | 2 (0.6) |
| Confusional state | 2 (0.6) |
| Osteoarthritis | 1 (0.3) |
| Colon cancer | 1 (0.3) |
| Acoustic neuroma | 1 (0.3) |
| Atrial fibrillation | 1 (0.3) |
| Breast cancer | 1 (0.3) |
| Cardiac failure | 1 (0.3) |
| Cholecystitis | 1 (0.3) |
| Cholecystitis acute | 1 (0.3) |
| Femur fracture | 1 (0.3) |
| Gastroesophageal reflux disease | 1 (0.3) |
| Intervertebral disc protrusion | 1 (0.3) |
| Parkinson’s disease | 1 (0.3) |
| Pyrexia | 1 (0.3) |
| Renal mass | 1 (0.3) |
| Sepsis | 1 (0.3) |
| Palpitations | 1 (0.3) |
| Indication and pre-treatment status refers to the primary treated eye. Only SAEs occurring during the safety observation period are included. Preferred terms are presented by descending order of frequency. A patient with multiple occurrences of a SAE was counted once per preferred term. A patient with multiple SAEs is counted only once in the total row. Patients with a baseline visit date present are included. Data collected until the last recorded follow-up date was used to perform the analyses.  CRVO, central retinal vein occlusion; n, number of patients; SAE, serious adverse events | |

**Supplementary Table 4. The LUMINOUS study principal investigators.**

| **Principal Investigator** | **Address** |
| --- | --- |
| **Argentina** | |
| Dr. Alejandro Cuomo | Clinica Modelo de Lanus, Lanus, Buenos Aires, 1824, Argentina |
| Dr. Alejandro Ferrero | Clínica de Ojos Srl, Santa Fe, Santa Fe, S3000FQI, Argentina |
| Dr. Ana Brant | Clínica Privada de Ojos José León Suárez, San Martin, Buenos Aires, 1650, Argentina |
| Dr. Andres Jacofsky | Consultorio Dr. Andres Jakofsky, Buenos Aires, Buenos Aires, C1122AAI, Argentina |
| Dr. Carlos Petry | Oftalmologia Integral, Mar del Plata, Buenos Aires, B7600, Argentina |
| Dr. David Pelayes | Consultorio de Investigaciones Oftalmológicas, Buenos Aires, Buenos Aires, 1425, Argentina |
| Dr. Federico Furno Sola | Grupo laser Visión - Rosario Eximer Laser Visión, Rosario, Santa Fe, S2000DLA, Argentina |
| Dr. Gustavo Fugazzotto | Consultorios Oftalmologico Dres Fugazzotto, Guaymallén, Mendoza, M5500, Argentina Plaza Vision S.A., Mendoza, Mendoza, M5500, Argentina |
| Dr. Gustavo Russo | Hospital Oftalmológico Malvinas Argentinas, Malvinas Argentinas, Buenos Aires, 1846, Argentina |
| Dr. Hugo Sudria | Instituto Oftamológico de Córdoba SA, Cordoba, Cordoba, 5000, Argentina |
| Dr. Matko Vidosevich | Microcirugia Ocular (Clínica MICRO), Rosario, Santa Fe, S2000CTC, Argentina |
| Dr. Mauricio Martinez Cartier | Instituto de la Visión, Ciudad Autonoma Buenos Aires, Ciudad Autonoma Buenos Aires, C1122, Argentina |
| Dr. Meroni Mariano | Clínica Oftalmológica Meroni, La Plata, Buenos Aires, B1900, Argentina |
| Dr. Nelida Rosso Nano | Clínica de Ojos, Concepción del Uruguay, Entre Rios, CP 3260, Argentina |
| Dr. Noe Rivero Covre | Clínica Dr Rivero Covre, Villa Gobernador Galvez, Santa Fe, 2124, Argentina |
| Dr. Pablo Ventola | Centro Oftalmologico Ventola, Caballito, Buenos Aires, C1437AVJ, Argentina |
| Dr. Paula Donato | Instituto Donato, Ciudad Autonoma Buenos Aires, Ciudad Autonoma Buenos Aires, 1428, Argentina |
| Dr. Pedro Miranda | Dyter S.A., Mendoza, Mendoza, 5500, Argentina |
| **Australia** | |
| Dr. Alex Hunyor | Retina Associates - Chatswood Retina Service, Chatswood, New South Wales, 2067, Australia |
| Prof. Andrew Chang | Sydney Retina Eye Clinic and Day Surgery, Sydney, New South Wales, 2000, Australia |
| Dr. Anthony Dunlop | Forster Eye Surgery, Forster, New South Wales, 2428, Australia |
| Prof. Anthony Kwan | Queensland Eye Institute, South Brisbane, Queensland, 4101, Australia |
| Dr. Brendan Vote | Tasmanian Eye Institute, South Launceston, Tasmania, 7249, Australia |
| Dr. Erwin Groeneveld | Brisbane Eye Clinic, Spring Hill, Queensland, 4000, Australia |
| Prof. Grant Raymond | Eye Consultants SA, North Adelaide, 5006, Australia |
| Prof. Ian McAllister | Lions Eye Institute, Nedlands, 6009, Australia |
| Dr. Jagjit Gilhotra | Adelaide Eye and Retina Centre, Adelaide, South Australia, 5000, Australia |
| Dr. James Wong | Strathfield Retina Clinic, Sydney, New South Wales, 2135, Australia |
| Dr. Jennifer Arnold | Marsden Eye Specialists, Sydney, New South Wales, 2150, Australia |
| Dr. John Clark | St. John of God Hospital, Geelong, Victoria, 3220, Australia |
| Prof. Mark Gillies | Sydney Eye Hospital, Sydney, New South Wales, 2000, Australia |
| Dr. Mark Gorbatov | Retina and Vitreous Centre, Strathfield, New South Wales, 2135, Australia |
| Dr. Mark Steiner | Oakleigh Eye Center, Oakleigh, Victoria, 3166, Australia |
| Dr. Nitin Verma | Hobart Eye Surgeions, Hobart, 5000, Australia |
| Dr. Paul Beaumont | Retina & Vitreous Centre, Sydney, New South Wales, 2000, Australia |
| Prof. Paul Mitchell | Private Rooms, Eye Clinic (B4a) Westmead Hospital, Westmead, New South Wales, 2145, Australia |
| Dr. Robert Bourke | Vision Retinal Institute, Southport, Queensland, 4215, Australia |
| Dr. Robert Chong | Southern Ophthalmology, Sydney, New South Wales, 2217, Australia |
| Prof. Robin Guymer | Macular Research Unit, Centre for Eye Research, East Melbourne, Victoria, 3002, Australia |
| Dr. Rohan Merani | Macquarie University, Sydney, New South Wales, 2109, Australia |
| Prof. Simon Chen | Vision Eye Institute Chatswood, Chatswood, 2067, Australia |
| Dr. Vivek Phakey | Waverley Eye Clinic, Glen Waverley, Victoria, 3150, Australia |
| Dr. William Campbell | Melbourne Retina Associates, East Melbourne, Victoria, 3002, Australia |
| **Austria** | |
| Prof. Siegfried Priglinger | Prof. Dr. Siegfried Priglinger, Linz, 4020, Austria |
| Prof. Ursula Schmidt-Erfurth | General University Hospital of Vienna, Vienna, Vienna, 1090, Austria |
| **Belgium** | |
| Prof. Anita Leys | Academic Hospital St. Rafaël, Leuven, 3000, Belgium |
| Dr. Danielle Vangermeersch | Imeldaziekenhuis, Bonheiden, 2820, Belgium |
| Prof Elisabeth Van Aken | Sint-Elisabeth Ziekenhuis, Zottegem, 9620, Belgium |
| Dr. Els Mangelschots | Virga Jesseziekenhuis, Hasselt, 3500, Belgium |
| Dr. Filip Mergaerts | RZ Tienen Campus St Elisabeth Aarschot, Aarschot, 3201, Belgium |
| Dr. Florence Rasquin | Hôpital Erasme, Bruxelles, 1070, Belgium |
| Dr. Guy Sallet | Ooginstituut, Aalst, 9300, Belgium |
| Dr. Ingrid Van der Donck | General Hospital Heilige Familie, Aarschot, 2840, Belgium |
| Dr. Isabelle Verhaeghe | General Hospital Maria Middelares, Ghent, 9000, Belgium |
| Dr. Jan Van Looveren | UZ Antwerpen, Edegem, 2650, Belgium |
| Dr. Laurent Levecq | Cliniques Universitaires de Mont-Godinne, Yvoir, B-5530, Belgium |
| Dr. Luana Bistreanu | Centre Hospitalier de Mouscron, Mouscron, 7700, Belgium |
| Dr. Stefano Barile | Centre Hospitalier Régional de Namur, Namur, 5000, Belgium |
| Dr. Thierry Vandercam | Centre Hospitalier de Dinant, Dinant, 5500, Belgium |
| **Brazil** | |
| Dr. Aderbal de Albuquerque Alves Junior | HFSE - Hospital Federal dos Servidores do Estado do Rio de Janeiro, Rio de Janeiro, 20221, Brazil |
| Dr. Arnaldo Bordon | ANGIOCORPORE, Sao Paulo, 040 21, Brazil |
| Dr. Danielle Lavinsky | Clínica Lavinsky Oftalmologia, Porto Alegre, Rio Grande do Sul, 90440, Brazil |
| Dr. Marcio Nehemy | Instituto da Visão, Belo Horizonte, Minas Gerais, 30150-270, Brazil |
| Dr. Marcos Pereira de Avila | CBCO - Centro Brasileiro de Cirurgia de Olhos, Goiânia, Goiás, 74210, Brazil |
| Dr. Osias de Souza | Centro Medico de Oftalmologia, Cambui, Campinas, 13092, Brazil |
| **Canada** | |
| Dr. Arif Samad | Ophthalmology Private Practice Dr Samad, Halifax, Nova Scotia, B3H 1Y6, Canada |
| Dr. Berhard Hurley | The Ottawa Hospital - General Campus, University of Ottawa Eye Institute, Ottawa, Ontario, K1H 8L6, Canada |
| Dr. David Chow | Toronto Retina Institute, Don Mills, Ontario, M1E 4A7, Canada |
| Dr. Deepa Yoganathan | North Toronto Eye Care, Downsview, Ontario, M3N 2V6, Canada |
| Dr. Don Nixon | Trimed Eye Center, Barrie, Ontario, L4M 4S5, Canada |
| Dr. Geoff Williams | Calgary Retina Consultants, Calgary, Alberta, T2H 0C8, Canada |
| Dr. Jason Noble | Scarborough Eye Clinic, Scarborough, Ontario, M1R 3A6, Canada |
| Dr. Laurent Lalonde | Institut de loeil des Laurentides, Boisbriand, Quebec, J7H 1S6, Canada |
| Dr. Luis Riveros | Miramichi Regional Hospital, Miramichi, New Brunswick, E1V 1N9, Canada |
| Dr. Michael Brent | Toronto Western Hospital, Toronto, Ontario, M5T 2S8, Canada |
| Dr. Murray Erasmus | Retina Consultants of Victoria (RCV), Victoria, British Columbia, V8V 4X3, Canada |
| Dr. Patrick Saurel | Clinique Dr. Patrick Saurel, Drummondville, Quebec, J2C 2C4, Canada |
| Dr. Raman Tuli | The Retina Centre of Ottawa, Ottawa, Ontario, K1Z 8R2, Canada |
| Dr. Sanjay Sharma | The Macula Clinic at the Wedgewood, Brockville, Ontario, K6V 0A6, Canada The Macula Clinic at the Bayview Medical Clinic, Belleville, Ontario, K8N 1E6, Canada |
| Dr. Sohel Somani | EyeMD Institute Suite, Brampton, Ontario, L6Y 0P6, Canada |
| Dr. Steve Dorrepaal | Clarity Eye Institute, Vaughan, Ontario, L4K 0C5, Canada |
| Dr. Thomas Sheidow | SJHC St. Joseph's Hospital, London, Ontario, N6A 4V2, Canada |
| Dr. Vineet Arora | Vineet I Arora Medicine Professional Corporation, Hamilton, Ontario, L9C 5R3, Canada |
| **Chile** | |
| Dr. Alejandro Lutz Herrerra | Hospital del Cobre Salvador Allende Gossens, Calama, 1399001, Chile |
| **China** | |
| Prof. Fang Wang | No. 10 People's Hospital of Shanghai, Shanghai, 200072, China |
| Prof. Fangtian Dong | Peking Union Medical College Hospital, Beijing, 100032, China |
| Prof. Gezhi Xu | Eye and ENT hospital, Shanghai, 200000, China |
| Prof. Guanfang Su | No.2 Hospital Affiliated to Jilin University, Changchun City, Jilin, 130041, China |
| Prof. Guoji Wu | Xiamen Eye Centre, Fujian, 361001, China |
| Dr. Hong Dai | Beijing Hospital, Beijing, 100730, China |
| Prof. Hongsheng Bi | Shierming Eye Hospital, Jinan, Shandong, 250001, China |
| Prof. Libo Xiao | Yunnan 2nd People's Hospital, Kunming, Yun'nan, 650021, China |
| Prof. Lin Lu | Zhongshan Ophthalmic Center, Sun Yat-sen University, Guangzhou, Guangdong, 510060, China |
| Prof. Liu Yang | Peking University First Hospital, Beijing,P.R., Beijing, 100034, China |
| Prof. Peiquan Zhao | Xinhua Hospital Affiliated to Shanghai Jiao Tong University School of Medicine, Shanghai, 200092, China |
| Prof. Wenbin Wei | Beijing Tong Ren Hospital, Capital Medical University, Beijing, 100730, China |
| Prof. Xian Wang | The Affiliated Hospital of Guiyang Medical College, Guiyang, Guizhou, 500000, China |
| Prof. Xiaorong Li | Tianjin Medical University Eye Center, Tianjin, 300384, China |
| Prof. Xiaowei Gao | No. 474 Hospital of PLA, Wulumuqi, Xinjiang Uygur, 830000, China |
| Prof. Xun Xu | Shanghai First People's Hospital, Shangai, 200080, China |
| Prof. Yi Wang | Southwest Hospital, Chongqing, Chongqing, 400038, China |
| Prof. Yiqiao Xing | Renmin Hospital of Wuhan University, Wuhan, Hubei, 430060, China |
| Prof. Zhengqin Yin | Chinese PLA General Hospital, Beijing, Beijing, 100853, China |
| Prof. Zhizhong Ma | Peking University Third Hospital, Beijing, 100191, China |
| **Colombia** | |
| Dr. Andres Amaya | Sociedad de Cirugía Ocular, Bogotá, , Colombia |
| Dr. Carlos Velez | Clínica Oftalmológica del Atlántico, Barranquilla, , Colombia |
| Dr. Diego Fernando Paipilla | Optisalud SAS, Yopal, , Colombia |
| Dr. Javier Andres Bernal Urrego | Clinica Oftalmologica, Armenia, , Colombia |
| Dr. Juan Pablo Sinisterra | Instituto de Ciegos y Sordos INSORP, Cali, Colombia |
| Dr. Maria Teresa Bernal | Clínica Barraquer, Bogotá, , Colombia |
| **Costa Rica** | |
| Dr. Lihteh Wu | Instituto de Cirugia Ocular, San José, San José, Costa Rica |
| Dr. Manrique Ortiz | Oftalmocima, San José, San José, Costa Rica |
| Dr. Teodoro Evans | Clínica 20/20, San José, San José, Costa Rica |
| **Czech Republic** | |
| Dr. Hana Fidranska | Fakultni nemocnice Plzen, Plzen, 323 00, Czech Republic |
| Dr. Jan Ernest | Ustredni vojenska nemocnice Praha, Praha, 169 02, Czech Republic |
| Dr. Jan Nemcansky | University Hopsital Ostrava, Ostrava, 708 52, Czech Republic |
| Dr. Jan Studnicka | Fakultni nemocnice Hradec Kralove, Hradec Kralove, 500 05, Czech Republic |
| Dr. Jiri Rehak | Fakultni nemocnice Olomouc, Olomouc, 775 20, Czech Republic |
| Dr. Martina Zavorkova | Krajska zdravotni, a.s. - Masarykova nemocnice v Usti nad Labem, Usti nad Labem, 40113, Czech Republic |
| Dr. Miroslav Veith | Fakultni nemocnice Kralovske Vinohrady, Praha 10, 100 34, Czech Republic |
| Prof. Petr Kolar | Fakultni nemocnice Brno, Brno, 625 00, Czech Republic |
| Dr. Zora Dubska | Charles University Hopsital 1st Faculty of Medicine, Praha 2, 128 08, Czech Republic |
| **Dominican Republic** | |
| Dr. Niurka Leonor | Centro Cardio-Neuro Oftalmológico y Trasplante (CECANOT), Santo Domingo, Santo Domingo, Dominican Republic |
| Dr. Rosina Negrin | Consultorio Oftalmológico Medicalnet, Santo Domingo, Santo Domingo, Dominican Republic |
| **Ecuador** | |
| Dr. Mario Polit Macias | Centro Medico Quirurgico Oftalmologico Alta Vision, Guayaquil, 090 150, Ecuador |
| **Egypt** | |
| Dr. Islam Waly | Egypt Air Hospital, Cairo, Cairo, Egypt |
| Prof. Mohamed Mahgoub | Eye Subspeciality Center, Cairo, Cairo, Egypt |
| Prof. Noha Khater | Al Mouneer Diabetic Retina Center, Giza, Cairo, Egypt |
| Dr. Walid Sheta | Alex Eye Center, Alexandria, Alexandria, Egypt |
| Prof. Yasser Soliman | Al Rowad Hospital, Giza, Cairo, Egypt |
| **France** | |
| Dr. Alain Donati | Cabinet d'Ophtalmologie, Melun, Seine et Marne, 77000, France |
| Prof. Catherine Creuzot-Garcher | CHU Dijon - Hopital General, Dijon, Côte-d'Or, 21000, France |
| Dr. Catherine Favard | Centre Ophtalmologique de L'Odeon, Paris, 75006, France |
| Dr. Christian Delhay | Clinique Ocean, Vannes, Côte-d'Or, 56000, France |
| Prof. Eric Souied | Centre Hospitalier Intercommunal de Créteil, Creteil, Val de Marne, 94010, France |
| Dr. Franck Rumen | Visiopole Private Practice, Lagord, Charente, 17140, France |
| Prof Gabriel Coscas | Cabinet Odeon , Paris, 75006, France |
| Dr. Hassiba Oubraham | Clinique de Montargis, Montargis, Loiret, 45200, France |
| Prof. Jean-Francois Korobelnik | Groupe Hospitalier Pellegrin -Hôpital Pellegrin, Bordeaux, Gironde, 33000, France |
| Dr. Laurence Mahieu | CHU Toulouse, Hôpital Paule de Vignier, Toulouse, Cedex 9, 31059, France |
| Dr. Laurent Khaitrine | Polyclinique de Courlancy, Reims, Marne, 51100, France |
| Prof. Laurent Kodjikian | Centre Hospitalier de la Croix Rousse, Lyon, Rhone, 69317, France |
| Dr. Martine Faysse | Fondation Ophtalmologique Adolphe de Rothschild, Paris, 75019, France |
| Prof. Michel Weber | CHU Nantes - Hôtel Dieu, Nantes Cedex 1, Loire Atlantique, 44093, France |
| Dr. Ramin Tadayoni | Hôpital Lariboisière, Paris, 75475, France |
| Dr. Salomon-Yves Cohen | Centre Ophtalmologique d’Imagerie et de Laser, Paris, Val de Marne, 75015, France |
| Dr. Stephanie Baillif-Gostoli | CHU de Nice - Hôpital Lenval, Nice, Alpes Maritimes, 060 06, France |
| Dr. Vincent Gualino | Clinique Orl Honore Cave, Montauban, Tarn et Garonne, 82000, France |
| **Germany** | |
| Dr. Annette Grote-Schmidt | Private practice_ Dr Grote-Schmidt, Tönisvorst, Nordrhein Westfalen, 47918, Germany |
| Dr. Arthur Mueller | Klinikum Augsburg Augenklinik, Augsburg, Bayern, 86156, Germany |
| Dr. Barbara Fuchs-Koelwel | Dr. med. Claus Fuchs Fachärzte für Augenheilkunde, Regensburg, Bayern, 93047, Germany |
| Dr. David Schell | Augenlaserzentrum, Neu-Ulm, Bayern, 89231, Germany |
| Dr. Erik Beeke | Klinikum Osnabrück, Osnabrück, Niedersachsen, 49076, Germany |
| Dr. Eva-Maria Weinschrod | Fachärztin für Augenheilkunde, Rothenburg, Bayern, 91541, Germany |
| Prof. Focke Ziemssen | Eberhard Karls University Eye Hospital, Tuebingen, Baden Wuerttemberg, 72076, Germany |
| Prof. Frank Holz | University of Bonn, Bonn, Nordrhein Westfalen, 53127, Germany |
| Dr. Georg Spital | St. Franziskus Hospital, Muenster, Nordrhein Westfalen, 48145, Germany |
| Dr. Gunther Kahle | Praxis Arzt für Augenheilkunde, Berlin, 10707, Germany |
| Dr. Kathleen Steinberg | Praxis Steinberg, Berlin, 10367, Germany |
| Dr. Mark Steiner | Augenklinik Dannenberg, Dannenberg Elbe, Niedersachsen, 29451, Germany |
| Prof. Nicole Eter | Universitaetsklinikum Muenster, Münster, Nordrhein Westfalen, 48149, Germany |
| Dr. Peter Kaupke | Praxis_Dr Kaupke, Hamburg, 22587, Germany |
| Prof. Sascha Fauser | Universitaetsklinikum Koeln, Köln, Nordrhein Westfalen, 50924, Germany |
| Dr. Steffen Rabethge | Dr. Rabethge Klinik GmbH, Schriesheim, Baden Wuerttemberg, 69198, Germany |
| Dr. Susanne Kaskel-Paul | Klinikum Lüdenscheid, Lüdenscheid, Nordrhein Westfalen, 58515, Germany |
| Dr. Thomas Grasbon | Augenarztpraxis Grasbon, Ingolstadt, Bayern, 85049, Germany |
| **Greece** | |
| Dr. Aleksandros Charonis | Athens Vision Eye Institute, Athens, 17673, Greece |
| Dr. Aliki Liaska | General Hospital of Lamia, Lamia, 35100, Greece |
| Dr. Efstratios Parikakis | Ophtalmiatrio Athinon - Athens Eye Hospital, Athens, 10672, Greece |
| Dr. Emmanouil Christodoulakis | General Hospital of Rethymnon, Rethymnon, 74100, Greece |
| Prof. Evangelia Tsironi | University General Hospital of Larissa, Larissa, 41110, Greece |
| Dr. Ioannis Datseris | Omma Ophtalmological Institute of Athens, Athens, 11525, Greece |
| Dr. Miltiadis Tsilimbaris | University Eye Hospital of Heraklion, Heraklion, 71201, Greece |
| Prof. Nicolas Farmakakis | University Hopsital of Patras, Patras, 26504, Greece |
| Dr. Stamatia Xirou | Red Cross Hospital -2nd Clinic, Athens, 11526, Greece |
| Prof. Stavros Dimitrakos | General Hospital Papageorgiou, Thessaloniki, 56429, Greece |
| Prof. Vasilios Kozobolis | University General Hospital of Alexandroupolis, Alexandroupolis, 68100, Greece |
| Dr. Vaso Konstantinidou | Eye Hospital of Athens -1st Clinic, Athens, 10672, Greece |
| **Guatemala** | |
| Dr. Fernando Noriega | Clínica Oftalmológica Santa Clara, Guatemala, Guatemala, Guatemala |
| **Hong Kong** | |
| Dr. Timothy Lai | Hong Kong Eye Hospital, Kowloon, Hong Kong |
| **Hungary** | |
| Dr. Andras Berta | Debreceni Egyetem Klinikai Kozpont, Debrecen, 4032, Hungary |
| Dr. Andras Seres | Budapest Retina Associates, Budapest, 1133, Hungary |
| Dr. Andrea Facsko | Szegedi Tudomanyegyetem Szent-Gyorgyi Albert Klinikai Kozpont, Szeged, 6720, Hungary |
| Dr. Janos Nemeth | Semmelweis Egyetem, Budapest, 1085, Hungary |
| Dr. Zsolt Biro | Pecsi Tudomanyegyetem, Pecs, 7624, Hungary |
| **India** | |
| Dr. Aditya Kelkar | National Institute of Ophthalmology, Pune, Maharashtra, 411005, India |
| Dr. Alay Banker | Bankers Eye Institute, Ahmedabad, Gujarat, 380009, India |
| Dr. Narendran Venkatapathy | Aravind  Eye Hospital, Coimbatore, Tamilnadu, 641014, India |
| Dr. Raja Narayanan | L. V. Prasad Eye Institute, Hyderabad, Andhra Pradesh, 500034, India |
| Dr. Taraprasad Das | L. V. Prasad Eye Institute, Bhubaneswar, Orissa, 751024, India |
| Dr. Tarun Sharma | Sankara Nethralaya, Chennai, Tamilnadu, 600006, India |
| Dr. Yograj Sharma | All India Institute of Medical Sciences, New Delhi, Delhi, 110029, India |
| **Ireland** | |
| Mr. David Keegan | Mater Private Hospital, Dublin, Dublin, 7, Ireland |
| Dr. Emer Henry | Waterford Regional Hospital, Waterford, X91, Ireland |
| Dr. Mark Cahill | Beacon Clinic, Dublin, 18, Ireland |
| Prof. Stephen Beatty | Whitfield Clinic, Waterford, X91, Ireland |
| **Israel** | |
| Prof. Hanna Garzozi | Bnai Zion Medical Center, Haifa, 31048, Israel |
| Dr. Irit Rosenblatt | Rabin Medical Center-Beilinson Campus, Petach Tikva, 4941492, Israel |
| Dr. Michaella Goldstein | Tel Aviv Sourasky Medical Center, Tel Aviv, 64239, Israel |
| **Italy** | |
| Prof. Angelo Minnella | Policlinico Universitario Agostino Gemelli, Roma, 001 68, Italy |
| Dr. Bruno Falcomata | Azienda Ospedaliera Bianchi Melacrino Morelli, Reggio Calabria, 89100, Italy |
| Dr. Daniela Dolcino | Reparto diOculistica dell Ospedale SS Antonio e Biagio e Cesare Arrigo de Alessandria, Alessandria, 15121, Italy |
| Prof. Francesco Semeraro | Azienda Socio Sanitaria Territoriale degli Spedali Civili di Brescia (Presidio Spedali Civili), Brescia, 25123, Italy |
| Dr. Luigina Tollot | Ospedale San Martino di Belluno, Belluno, 32100, Italy |
| Prof. Marco Nardi | Azienda Ospedaliero Universitaria Cisanello, Pisa, 56126, Italy |
| Prof. Maurizio Fossarello | Ospedale S.Giovanni di Dio, Cagliari, 091 00, Italy |
| Dr. Rosalia Giustolisi | Umberto I Pol. di Roma-Universita di Roma La Sapienza, Roma, 001 61, Italy |
| Dr. Tommaso Micelli Ferrari | Ente Ecclesiastico Ospedale Generale Regionale F Miulli, Acquaviva delle Fonti, Bari, 70021, Italy |
| **Japan** | |
| Dr. Akira Arakawa | Yokohama City University Medical Center, Yokohama-shi, Kanagawa-Ken, 232-0024, Japan |
| Dr. Akira Obana | Seirei Hamamatsu General Hospital, Hamamatsu-shi, Shizuoka-Ken, 430-8558, Japan |
| Dr. Akiteru Kawahara | University of Miyazaki Hospital, Miyazaki-shi, Miyazaki-Ken, 889-1692, Japan |
| Prof. Atsushi Hayashi | Toyama University Hospital, Toyama-shi, Toyama-Ken, 930-0194, Japan |
| Dr. Atsushi Hirota | Hirota Eye Clinic, Shunan-shi, Yamaguchi-Ken, 745-0017, Japan |
| Dr. Atsushi Otani | Japan Red Cross Society Wakayama Medical Center, Wakayama-shi, Wakayama-Ken, 640-8558, Japan |
| Prof. Ayame Annabel Okada | Kyorin University Hospital, Mitaka-shi, Tokyo-To, 181-8611, Japan |
| Dr. Chieko Shiragami | Kagawa University Hospital, Kita-gun, Kagawa-Ken, 761-0793, Japan |
| Dr. Chota Matsumoto | Kindai University Hospital, Osakasayama-shi, Osaka-Fu, 589-8511, Japan |
| Dr. Daisuke Jin | Akita University Hospital, Akita-shi, Akita-Ken, 010-8543, Japan |
| Dr. Eiichi Sato | Asahikawa Medical University Hospital, Asahikawa-shi, Hokkaido, 078-8510, Japan |
| Dr. Eriko Matsushita | Kochi Medical School Hospital, Nankoku-shi, Kochi-Ken, 783-8505, Japan |
| Dr. Futoshi Ishikawa | Sapporo Medical University Hospital, Sapporo-shi, Hokkaido, 060-8543, Japan |
| Prof. Goji Tomita | Toho University Ohashi Medical Center, Meguro-ku, Tokyo-To, 153-8515, Japan |
| Dr. Goro Watanabe | Koyokai Yayoi Hospital, Toyohashi-shi, Aichi-Ken, 441-8106, Japan |
| Dr. Hajime Sato | JOHAS Tohoku Rosai Hospital, Sendai-shi, Miyagi-Ken, 981-8563, Japan |
| Dr. Harumi Wakiyama | Japanese Red Cross Nagasaki Genbaku Hospital, Nagasaki-shi, Nagasaki-Ken, 852-8511, Japan |
| Dr. Hidenori Takahashi | Jichi Medical University Hospital, Shimotsuke-shi, Tochigi-Ken, 329-0498, Japan |
| Prof. Hidetoshi Yamashita | Yamagata University Hospital, Yamagata-shi, Yamagata-Ken, 990-9585, Japan |
| Dr. Hideyasu O | Hyogo Prefectural Amagasaki General Medical Center, Amagasaki-shi, Hyogo-Ken, 660-8550, Japan |
| Dr. Hiroaki Kobayashi | Juntendo University Hospital, Bunkyo-ku, Tokyo-To, 113-8431, Japan |
| Dr. Hiroaki Ushida | Shizuoka Saiseikai General Hospital, Shizuoka-shi, Shizuoka-Ken, 422-8527, Japan |
| Dr. Hiroko Imaizumi | Sapporo City General Hospital, Sapporo-shi, Hokkaido, 060-8604, Japan |
| Dr. Hisashi Matsubara | Mie University Hospital, Tsu-shi, Mie-Ken, 514-8507, Japan |
| Dr. Ichiro Ota | Shozankai Miyake Eye Hospital, Nagoya-shi, Aichi-Ken, 462-0825, Japan |
| Dr. Jiro Kogo | St. Marianna University School of Medicine Hospital, Kawasaki-shi, Kanagawa-Ken, 216-8511, Japan |
| Dr. Jun Yamada | Meiji University of Integrative Medicine, Nantan-shi, Kyoto-Fu, 629-0392, Japan |
| Prof. Kanji Takahashi | Kansai Medical University Hospital, Hirakata-shi, Osaka-Fu, 573-1191, Japan |
| Dr. Kaori Sayanagi | Osaka University Hospital, Suita-shi, Osaka-Fu, 565-0871, Japan |
| Dr. Kazuaki Nishijima | Nishijima Eye Clinic, Kyoto-shi, Kyoto-Fu, 604-0837, Japan |
| Dr. Kazuhiko Dannoue | Dannoue Eye Clinic, Kawasaki-shi, Kanagawa-Ken, 211-0053, Japan |
| Dr. Kazunori Miyata | Meiwakai Miyata Ophthalmic Hospital, Miyakonojo-shi, Miyazaki-Ken, 885-0051, Japan |
| Dr. Kohei Ishikawa | Ishikawa Eye Clinic, Shizuoka-shi, Shizuoka-Ken, 420-0841, Japan |
| Prof. Koichi Ohta | Matsumoto Dental University Hospital, Shiojiri-shi, Nagano-Ken, 399-0781, Japan |
| Dr. Koji Aoyagi | Aoyagi Eye Clinic, Ueda-shi, Nagano-Ken, 386-0002, Japan |
| Dr. Kunihiro Musashi | Musashi Dream Eye Clinic, Osaka-shi, Osaka-Fu, 543-0027, Japan |
| Prof. Masahiko Shimura | Tokyo Medical University Hachioji Medical Center, Hachioji-shi, Tokyo-To, 193-0998, Japan |
| Dr. Masahiro Miura | Tokyo Medical University Ibaraki Medical Center, Inashiki-gun, Ibaraki-Ken, 300-0395, Japan |
| Prof. Masahito Ohji | Shiga University of Medical Science Hospital, Otsu-shi, Shiga-Ken, 520-2192, Japan |
| Dr. Masaru Inatani | University of Fukui Hospital, Yoshida-gun, Fukui-Ken, 910-1193, Japan |
| Prof. Masayuki Horiguchi | Fujita Health University Hospital, Toyoake-shi, Aichi-Ken, 470-1192, Japan |
| Dr. Michiko Takamiya | Yonezawa City Hospital, Yonezawa-shi, Yamagata-Ken, 992-8502, Japan |
| Dr. Misa Suzuki | Totsukaekimae Suzuki Eye Clinic, Yokohama-shi, Kanagawa-Ken, 244-0003, Japan |
| Dr. Mizuki Tagami | Kobe Kaisei Hospital, Kobe-shi, Hyogo-Ken, 657-0068, Japan |
| Dr. Nagahisa Yoshimura | Kyoto University Hospital, Kyoto-shi, Kyoto-Fu, 606-8507, Japan |
| Prof. Nahoko Ogata | Nara Medical University Hospital, Kashihara-shi, Nara-Ken, 634-8522, Japan |
| Dr. Naotaka Kanda | Jusendo General Hospital, Koriyama-shi, Fukushima-Ken, 963-8585, Japan |
| Dr. Naoyasu Umeda | Fukuoka University Hospital, Fukuoka-shi, Fukuoka-Ken, 814-0180, Japan |
| Dr. Rei Ito | Juntendo University Urayasu Hospital, Urayasu-shi, Chiba-Ken, 279-0021, Japan |
| Dr. Rumiko Hara | Kakogawa City West Hospital, Kakogawa-shi, Hyogo-Ken, 675-8611, Japan |
| Dr. Ryo Eguchi | Ando Eye Clinic, Ashigarakami-gun, Kanagawa-Ken, 258-0003, Japan |
| Dr. Ryoichi Sugawara | Kitami Red Cross Hospital, Kitami-shi, Hokkaido, 090-8666, Japan |
| Dr. Ryoko Okayama | Saiando Ochanomizu Inoue Eye Clinic, Chiyoda-ku, Tokyo-To, 101-0062, Japan |
| Dr. Ryoko Osawa | Kaiya Ophthalmology, Hamamatsu-shi, Shizuoka-Ken, 430-0903, Japan |
| Dr. Ryusaburo Mori | Nihon University Hospital, Chiyoda-ku, Tokyo-To, 101-8309, Japan |
| Dr. Sakura Sato | Taidokai Sato Ganka Iin Domachi Clinic, Yamagata-shi, Yamagata-Ken, 990-0051, Japan |
| Dr. Santoshi Takeuchi | Yokosuka Kyosai Hospital, Yokohama-shi, Kanagawa-Ken, 236-0004, Japan |
| Dr. Shigeki Tagawa | Tagawa Eye Clinic, Kanazawa-shi, Ishikawa-Ken, 920-1151, Japan |
| Dr. Shigeki Yamanishi | Matsuyama Red Cross Hospital, Matsuyama-shi, Ehime-Ken, 790-8524, Japan |
| Dr. Shigeru Hoshiai | Hoeikai Hoshiai Eye Clinic, Saitama-shi, Saitama-Ken, 336-0963, Japan |
| Dr. Shigeto Fujimura | Kanazawa University Hospital, Kanazawa-shi, Ishikawa-Ken, 920-8641, Japan |
| Dr. Shin Yoneya | Saitama Medical University Hospital, Iruma-gun, Saitama-Ken, 350-0495, Japan |
| Dr. Shinichiro Otani | Meiwakai Kagoshima Miyata Ophthalmic Hospital, Kagoshima-shi, Kagoshima-Ken, 890-0046, Japan |
| Dr. Shinichiro Yoshida | Yoshida Eye Hospital, Hakodate-shi, Hokkaido, 041-0851, Japan |
| Dr. Shoji Kishi | Gunma University Hospital, Maebashi-shi, Gunma-Ken, 371-8511, Japan |
| Dr. Tadayuki Nishide | Yokohama City University Hospital, Yokohama-shi, Kanagawa-Ken, 236-0004, Japan |
| Dr. Taiichi Hikichi | Shuhokai Ohtsuka Eye Hospital, Sapporo-shi, Hokkaido, 001-0016, Japan |
| Prof. Taiji Sakamoto | Kagoshima University Medical And Dental Hospital, Kagoshima-shi, Kagoshima-Ken, 890-8520, Japan |
| Dr. Takahiro Uda | Ehime University Hospital, Toon-shi, Ehime-Ken, 791-0295, Japan |
| Dr. Takashi Fujishiro | Saitama Red Cross Hospital, Saitama-shi, Saitama-Ken, 338-8553, Japan |
| Prof. Takashi Kitaoka | Nagasaki University Hospital, Nagasaki-shi, Nagasaki-Ken, 852-8501, Japan |
| Dr. Takatomo Miyake | Ogaki Tokushukai Hospital, Ogaki-shi, Gifu-Ken, 503-0015, Japan |
| Prof. Taktoshi Maeno | Toho University Sakura Medical Center, Sakura-shi, Chiba-Ken, 285-8741, Japan |
| Dr. Taku Ogura | Japanese Red Cross Society Suwa Hospital, Suwa-shi, Nagano-Ken, 392-8510, Japan |
| Dr. Tatsuo Nagata | University of Occupational and Environmental Health Hospital, Kitakyushu-shi, Fukuoka-Ken, 807-8556, Japan |
| Prof. Tatsuro Ishibashi | Kyushu University Hospital, Higashi-ku, Fukuoka, 812-8582, Japan |
| Dr. Tatsushi Kaga | JCHO Chukyo Hospital, Nagoya-shi, Aichi-Ken, 457-8510, Japan |
| Prof. Tetsuju Sekiryu | Fukushima Medical University Hospital, Fukushima-shi, Fukushima-Ken, 960-1295, Japan |
| Prof. Tetsuro Oshika | Tsukuba University Hospital, Tsukuba-shi, Ibaraki-Ken, 305-8576, Japan |
| Dr. Tomohiro Iida | Tokyo Women's Medical University Hospital, Shinjuku-ku, Tokyo-To, 162-8666, Japan |
| Prof. Tomohiro Ikeda | Hyogo College of Medicine Hospital, Nishinomiya-shi, Hyogo-Ken, 663-8501, Japan |
| Dr. Tomoki Sakuraba | Aomori Prefectural Central Hospital, Aomori, Aomori, 030-8553, Japan |
| Dr. Tomoko Kawamura | Steel Memorial Hirohata Hospital, Himeji-shi, Hyogo-Ken, 671-1122, Japan |
| Dr Toru Nakazawa | Tohoku University Hospital, Sendai-shi, Miyagi-Ken, 980-8574, Japan |
| Prof. Toshihiko Ohta | Juntendo University Shizuoka Hospital, Izunokuni-shi, Shizuoka-Ken, 410-2295, Japan |
| Dr. Toshiyuki Yokoyama | Juntendo University Nerima Hospital, Nerima-ku, Tokyo-To, 177-8521, Japan |
| Dr. Toyohisa Yoshizawa | Jigankai Sanjo Eye Clinic, Sanjo-shi, Niigata-Ken, 955-0852, Japan |
| Dr. Tsuyoshi Otsuji | Kansai Medical University Takii Hospital, Moriguchi-shi, Osaka-Fu, 570-8507, Japan |
| Dr. Yasuki Ito | Nagoya University Hospital, Nagoya-shi, Aichi-Ken, 466-8560, Japan |
| Dr. Yoichi Sakurada | University of Yamanashi Hospital, Chuo-shi, Yamanashi-Ken, 409-3898, Japan |
| Dr. Yoshiaki Kiuchi | Hiroshima University Hospital, Hiroshima-shi, Hiroshima-Ken, 734-8551, Japan |
| Dr. Yoshihiro Hashimoto | Shinseikai Toyama Hospital, Imizu-shi, Toyama-Ken, 939-0243, Japan |
| Dr. Yoshihiro Wakabayashi | Tokyo Medical University Hospital, Shinjuku-ku, Tokyo-To, 160-0023, Japan |
| Prof. Yoshinori Mitamura | Tokushima University Hospital, Tokushima-shi, Tokushima-Ken, 770-8503, Japan |
| Dr. Yoshiyuki Kondo | Infinity Medical Group Kondo Eye Clinic, Hachioji-shi, Tokyo-To, 192-0081, Japan |
| Dr. Yukihiko Shiraki | Aichi Medical University Hospital, Nagakute-shi, Aichi-Ken, 480-1195, Japan |
| Dr. Yukihiro Horie | Obihiro Kyokai Hospital, Obihiro-shi, Hokkaido, 080-0805, Japan |
| Dr. Yuzo Suda | Dokkyo Medical University Hospital, Shimotsuga-gun, Tochigi-Ken, 321-0293, Japan |
| **Korea (South Korea)** | |
| Dr. Hakyoung Kim | Hallym University Kangnam Sacred Heart Hospital, Seoul, 150-950, Korea (Republic of Korea) |
| Dr. Hum Chung | Seoul National University Hospital, Seoul, Gyeonggi-do, 110744, Korea (Republic of Korea) |
| Prof. Hyoung Jun Koh | Gangnam Severance Hospital, Yonsei University Health System, Seoul, 062 73, Korea (Republic of Korea) |
| Dr. Hyun Woong Kim | Inje University Busan Paik Hospital, Busan, 47392, Korea (Republic of Korea) |
| Dr. Hyung-Woo Kwak | Kyung Hee University Hospital, Seoul, 024 47, Korea (Republic of Korea) |
| Dr. In Taek Kim | Kyungpook National University Hospital, Daegu, Gyeongsangbuk-do, 700-721, Korea (Republic of Korea) |
| Dr. Ji Eun Lee | Pusan National University Hospital, Busan, 602-739, Korea (Republic of Korea) |
| Dr. Ji Hun Song | Ajou University Hospital, Suwon-si, Gyeonggi-do, 16499, Korea (Republic of Korea) |
| Dr. Jong Woo Kim | Kim's Eye Hospital, Seoul, 150-034, Korea (Republic of Korea) |
| Dr. June-Gone Kim | Asan Medical Center, Seoul, 055 05, Korea (Republic of Korea) |
| Dr. Kyu Hyung Park | Seoul National University Bundang Hospital, Seongnam-si, Gyeonggi-do, 13620, Korea (Republic of Korea) |
| Dr. SeWoong Kang | Samsung Medical Center, Seoul, 063 51, Korea (Republic of Korea) |
| Dr. Won Ki Lee | The Catholic University of Korea, Seoul St. Mary’s Hospital, Seoul, 137-701, Korea (Republic of Korea) |
| Dr. Woohyok Chang | Yeungnam University Hospital, Daegu, 42415, Korea (Republic of Korea) |
| **Malaysia** | |
| Dr. Barkeh Hanim Bt Jumaat | International Specialist Eye Centre, Kuala Lumpur, Kuala Lumpur, 59200, Malaysia |
| Dr. Mae-Lynn Catherine Bastion | Pusat Perubatan Universiti Kebangsaan Malaysia, Kuala Lumpur, Kuala Lumpur, 56000, Malaysia |
| Dr. Nor Fariza Ngah | Hospital Selayang, Batu Caves, Selangor, 68100, Malaysia |
| Prof. Visvaraja Subrayan | University of Malaya Eye Research Centre, Kuala Lumpur, Kuala Lumpur, 50603, Malaysia |
| **Mexico** | |
| Dr. Abel Ramirez Estudillo | Hospital Oftalmológico Nuestra Señora de la Luz, Mexico City, Distrito Federal, 060 30, Mexico |
| Dr. Arturo Enriquez Huerta | Clínica de Ojos Monterrey S.A. de C.V., Monterrey, Nuevo León, 64060, Mexico |
| Dr. Ermilo Sanchez Buenfil | RetimediQ Centro de Retina y Oftalmologia Especializada, Merida, Yucatán, 97130, Mexico |
| Dr. Jesús González Cortez | Hospital Universitario Dr Jose E Gonzalez, Monterrey, Nuevo León, 64460, Mexico |
| Dr. Jose Dalma Weiszhausz | Dr. Alejandro Dalma y asoc., Mexico City, Distrito Federal, 11000, Mexico |
| Dr. Patricio Rodriguez Valdez | CIIES, Monterrey, Nuevo León, 64710, Mexico |
| Dr. René Cano Hidalgo | Instituto de Oftalmología Fundación Conde de la Valenciana, Mexico City, Distrito Federal, 068 00, Mexico |
| **Netherlands** | |
| Dr. Anne-Minke de Boer | OMC Amsterdam, Amsterdam, 1071NT, Netherlands |
| Dr. Janneke van Lith-Verhoeven | Sint Elisabeth Ziekenhuis Afd. Oogheelkunde, Tilburg, 5022 GC, Netherlands |
| Dr. Jenny Onkosuwito | Flevoziekenhuis, Almere, 1315 RA, Netherlands |
| **Panamá** | |
| Dr. Ana Paz | Clínica de Vitreo y Macula Dra. Ana Paz, Panamá, Panamá, Panamá |
| Dr. Roberto Yee | Clinica Yee, Panamá, Panamá, Panamá |
| **Peru** | |
| Dr. Giovanna Gonzalez Luey | Hospital Nacional Guillermo Almenara Irigoyen, Lima, 4559, Peru |
| Dr. Karen Barraza Lino | Instituto Oftalmosalud S.R.L, San Isidro, 15036, Peru |
| Dr. Miguel Guzman | Ophtalmology-TG Laser Oftalmica , Lima, 666, Peru |
| Dr. Silvio Lujan Najar | Macula D&T, San Isidro, 15036, Peru |
| **Poland** | |
| Dr. Ewa Fluder | Szpital Specjalistyczny im Sokołowskiego, Walbrzych, 58-300, Poland |
| Dr. Halina Wykrota | NZOZ Lens-Med, Katowice, 40-064, Poland |
| Dr. Ilona Pawlicka | Wojewódzki Szpital Okulistyczny w Krakowie, Krakow, 31-723, Poland |
| Dr. Jan Kucharczuk | 10 Wojskowy Szpital Kliniczny, Bydgoszcz, 85-681, Poland |
| Dr. Janusz Cieslik | NZOZ Medilens, Kielce, 25-514, Poland |
| Dr. Jerzy Mackiewicz | Medical University of Lublin, Lublin, 20-079, Poland |
| Prof. Krystyna Raczynska | Specjalistyczny Cabinet Lekarski Krystyna Raczynska, Gdansk, 80-147, Poland |
| Dr. Maciej Gawęcki | Szpital Specjalistyczny IM J.K. Lukowicza, Chojnice, 89-600, Poland |
| Dr. Maciej Gwóźdź | Samodzielny Publiczny ZOZ, Wolomin, 05-200, Poland |
| Dr. Małgorzata Figurska | Wojskowy Instytut Medyczny, Warszawa, 04-141, Poland |
| Mikołaj Meller | NZOZ Ocu Service, Poznan, 60-538, Poland |
| Dr. Piotr Gozdek | Szpital Zakonu Bonifratrów im. Św. Jana Bożego w Łodzi, Łódź, 93-357, Poland |
| Dr. Piotr Oleksy | Centrum Medyczne Uno-Med (Private Practice), Kalisz, 62-800, Poland |
| Dr. Sławomir Zalewski | Centrum Diagnostyki i Mikrochirurgii Oka LENS, Olsztyn, 10-424, Poland |
| **Portugal** | |
| Dr. Carla Teixeira | Hospital Pedro Hispano, Matosinhos, 4454-509, Portugal |
| Dr. João Chibante | Centro Hospitalar de Entre o Douro e Vouga, E.P.E - Hospital de São Sebastião, Santa Maria da Feira, 4520-211, Portugal |
| Dr. João Paulo Castro de Sousa | Centro Hospitalar Leiria - Hospital Santo André, Leiria, 2410-197, Portugal |
| Prof. José Cunha-Vaz | AIBILI, Coimbra, 3000-548, Portugal |
| Dr. Manuel Mariano | Centro Hospitalar do Baixo Vouga, E.P.E. – Unidade de Aveiro, Aveiro, 3814-501, Portugal |
| Dr. Rita Flores | HPP - Hospital dos Lusíadas, Lisbon, 1500-458, Portugal |
| Dr. Rufino Silva | Espaço Médico de Coimbra, Coimbra, 3030-163, Portugal |
| **Russia** | |
| Dr. Andrey Kuznetsov | MBHI City Clinical Hospital #11, Chelyabinsk, 454000, Russia |
| Prof. Andrey Shchuko | The Irkutsk Affiliate of Federal State Budgetary Institution "MNTK ye Microsurgery Complex" n.a. S.N, Irkutsk, 664033, Russia |
| Dr. Ekaterina Zakharova | Institution of Republic Sakha (Yakutiya) Yakutsk Republican Ophthalmology Hospital, Yakutsk, 677000, Russia |
| Dr. Elena Sumarokova | SBEI HPE "Saratov State Medical University n.a. V. I. Razumovskiy" of the MoH of the RF, Saratov, 410028, Russia |
| Dr. Elina Santoro | BI of Khanty-Mansyisk region Yugra "Surgut regional clinical hospital", Surgut, 628400, Russia |
| Dr. Elmira Abdulaeva | SAIH "Republican clinical ophthalmological hospital of MoH of Rebublic of Tatarstan", Kazan, 420012, Russia |
| Prof. Evgeniy Sergeevich Miludin | SBEI HPE "Samara State Medical University" of the MoH of the RF, Samara, 443099, Russia |
| Dr. Fedor Yevgenyevitch Shadrichev | Territorial Diabetic Center, Saint Petersburg, 194354, Russia |
| Dr. Galina Bratko | The S.N.Fyodorov Federal State Institution Eye Microsurgery Complex (Novosibirsk), Novosibirsk, 630071, Russia |
| Dr. Irina Mikhaylovna Rybina | Tyumen Regional Ophthalmology Dispensary, Tyumen, 625048, Russia |
| Dr. Lyubov Petrovna Danilova | The S.N.Fyodorov Federal State Institution Eye Microsurgery Complex (Khabarovsk), Khabarovsk, 680033, Russia |
| Dr. Maria Viktorovna Budzinskaya | Scientific Research Institute of Eye Diseases, Moscow, 119021, Russia |
| Dr. Marina Zimina | Chita State Medical Academy, Chita, 672090, Russia |
| Dr. Mukhtarram Mukhammarovitch Bikbov | SBI "Ufa scientific research institute of eye diseases of academy of sciences of the republic of Bas, Ufa, 450077, Russia |
| Dr. Nikolai Petrovitch Pashtaev | The S.N.Fyodorov Federal State Institution Eye Microsurgery Complex (Cheboxary), Cheboksary, 428028, Russia |
| Dr. Oleg L'vovich Fabrikantov | The S.N.Fyodorov Federal State Institution Eye Microsurgery Complex (Tambov), Tambov, 392000, Russia |
| Dr. Olga Vladimirovna Sarygina | Moscow Helmholtz Research Institute of Ophthalmology, Moscow, 105062, Russia |
| Dr. Sergey Sachnov | Krasnodar Branch of The S.N. Fyodorov FSBI "Eye microsurgery complex", Krasnodar, 350012, Russia |
| Dr. Timur Galeev | SBHI "Penza Regional Ophtalmological Hospital", Penza, 440026, Russia |
| Dr. Vitaly Sokolov | Dignostic Center №7, Saint Petersburg, 190000, Russia |
| **Saudi Arabia** | |
| Dr. Ammar Dawalibi | Prince Sultan Military Medical City, Riyadh, 11159, Saudi Arabia |
| Dr. Hassan Dhibi | King Khaled Eyes Specialist Hospital, Riyadh, 11462, Saudi Arabia |
| Dr. Karim Talaat | King Khalid National Guard Hospital, Jeddah, 21423, Saudi Arabia |
| Dr. Osama Alem | King Fahad National Guard Hospital, Riyadh, 14611, Saudi Arabia |
| **Singapore** | |
| Dr. Adrian Koh | Camden Medical Center, Singapore, 248649, Singapore |
| Dr. Gemmy Cheung | Singapore National Eye Centre, Singapore, 168751, Singapore |
| Prof. Tien Yin Wong | National University Hospital, Singapore, 119074, Singapore |
| **Slovakia** | |
| Dr. Alena Zencarova | Fakultna nemocnica s poliklinikou Nove Zamky, Nové Zámky, 940 01, Slovakia |
| Dr. Blandina Lipkova | Fakultna nemocnica s poliklinikou Zilina, Zilina, 010 01, Slovakia |
| Dr. Hedviga Miková | Nemocnica svateho Michala, Bratislava, 833 31, Slovakia |
| Dr. Jaroslav Hasa | Univerzitna nemocnica Bratislava, Nemocnica Ruzinov, Bratislava, 82606, Slovakia |
| Dr. Juraj Bajacek | Ustredna vojenska nemocnica SNP Ruzomberok- Fakultna nemocnica, Ruzomberok, 034 26, Slovakia |
| Dr. Livia Javorska | Nemocnica Poprad a.s., Poprad, 058 01, Slovakia |
| Dr. Maria Hurcikova | Nemocnica s poliklinikou Trebisov a.s., Trebišov, 075 01, Slovakia |
| Dr. Marta Ondrejkova | NsP Banská Bystrica, Banská Bystrica, 974 01, Slovakia |
| Dr. Monika Gajdosova | Oftal s.r.o., Zvolen, 960 01, Slovakia |
| Dr. Zuzana Jamrichova | Univerzitna nemocnica Bratislava, Nemocnica sv. Cyrila a Metoda, Bratislava, 851 07, Slovakia |
| Dr. Zuzana Sustykevicova | Fakultna nemocnica Trencin, Trenčín, 91101, Slovakia |
| **Slovenia** | |
| Dr. Ivana Gardaševič | General Hospital Novo Mesto, Novo Mesto, 8000, Slovenia |
| Dr. Marko Vrhovec | General Hospital Celje, Celje, 3000, Slovenia |
| Dr. Petra Skitek | University Medical Centre Maribor, Maribor, 2000, Slovenia |
| Dr. Polona Mekjavic | Univerzitetni Klinicni Center Ljubljana Ocesna Klinika, Ljubljana, 1000, Slovenia |
| **Spain** | |
| Dr. Belen Aurora Fente Sampayo | Hospital Universitario Lucus Augusti, Lugo, 27003, Spain |
| Dr. Emiliano Hernandez Galilea | Hospital Universitario de Salamanca, Salamanca, 37007, Spain |
| Dr. Enrique Lopez-Sanchez | Hospital Arnau de Vilanova, Valencia, 46015, Spain |
| Dr. Francisco Hurtado Cena | Clinica Rementeria, Madrid, 28010, Spain |
| Dr. Isabel Pinilla | Hospital Clinico Universitario Lozano Blesa, Zaragoza, 50009, Spain |
| Dr. Jesus Garcia Martinez | Hospital Moncloa, Madrid, 28008, Spain |
| Dr. Jose Buil Calvo | Hospital de la Santa Creu i Sant Pau, Barcelona, 080 25, Spain |
| Dr. Jose Escobar Barranco | Hospital Dos de Maig, Barcelona, 080 25, Spain |
| Dr. Juan Donate | Hospital Universitario Clinico San Carlos, Madrid, 28040, Spain |
| Dr. Laura Sararols | Fundacio Privada Hospital Asil de Granollers, Granollers, Barcelona, 084 02, Spain |
| Dr. Laura Sararols Ramsay | Hospital General de Catalunya, Sant Cugat del Valles, Barcelona, 8195, Spain |
| Dr. Lorenzo Lopez Guajardo | Hospital Universitario Principe de Asturias, Alcala de Henares, Madrid, 28805, Spain |
| Dr. Luis Moreno | Hospital Universitario 12 de Octubre, Madrid, 28041, Spain |
| Dr. Luis Pablo Julvez | Hospital Universitario Miguel Servet, Zaragoza, 50009, Spain |
| Dr. Manuel Franco Benito | Hospital Universitario de Leon, León, 24008, Spain |
| Dr. Manuel Gurrea | Centro Oftalmologico Gaztambide, Madrid, 28015, Spain |
| Dr. Manuela Contreras | Hospital Regional Universitario de Malaga, Málaga, 29010, Spain |
| Dr. Maribel Lopez-Galvez | Universidad de Valladolid, Valladolid, 47011, Spain |
| Dr. Marta Figueroa | Vissum Corporación Oftalmológica – Mirassierra, Madrid, 28035, Spain |
| Dr. Nuria Maria Gajate Paniagua | Hospital Universitario de Burgos, Burgos, 090 05, Spain |
| Dr. Pedro Aroca | Hospital Universitario Sant Joan de Reus, Reus, Tarragona, 431204, Spain |
| Dr. Roberto Gallego-Pinazo | Hospital Universitari i Politecnic La Fe, Valencia, 46026, Spain |
| Dr. Santiago Abengoechea | Centro de Oftalmología Barraquer, Barcelona, 8017, Spain |
| Dr. Sara Velilla Oses | Hospital San Pedro, Logrono, La Rioja, 26006, Spain |
| **Turkey** | |
| Prof. Berati Hasanreisoğlu | Gazi University Hospital, Ankara, 0 6560, Turkey |
| Prof. Bora Eldem | Hacettepe University Medical Faculty, Ankara, 0 6100, Turkey |
| Prof. Emin Ozmert | Ankara University Medical Faculty, Ankara, 0 6100, Turkey |
| Prof. Faruk Ozturk | Ankara Ataturk Training and Research Hospital, Ankara, 0 6100, Turkey |
| Dr. Rifat Rasier | Bilim University Florence Nightingale Hospital, Istanbul, 34387, Turkey |
| **Ukraine** | |
| Dr. Andriy Korol | V.P.Filatov Institute of Eye Diseases and Tissue Therapy AMS, Odesa, 65000, Ukraine |
| Dr. Andriy Petrunya | LLC “Lugansk Regional Central Eye Hospital”, Lugansk, 91055, Ukraine |
| Dr. Pavel Bezditko | Kharkov Regional Clinical Hospital, Kharkiv, 61022, Ukraine |
| Dr. Svjatoslav Suk | City clinical ophthalmological hospital, Kiev, 3065, Ukraine |
| Dr. Valerij Serdyuk | Regional clinical hospital n.a. Mechnikova, Dnipropetrovsk, 49027, Ukraine |
| **United Kingdom** | |
| Dr. Adam Booth | Queen Alexandra Hospital, Portsmouth, Hampshire, PO6 3LY, United Kingdom |
| Mr. Adam Ross | Bristol Eye Hospital (Retinal Treatment and Research Unit), Bristol, Avon, BS1 2LX, United Kingdom |
| Dr. Ahmed Kamal | University Hospital Aintree, NHS, Liverpool, Merseyside, L9 7AL, United Kingdom |
| Mr. Aires Lobo | Bedford Hospital,, Bedford, Bedfordshire, MK42 9DJ, United Kingdom |
| Prof. Andrew Lotery | Southampton General Hospital, Southampton, Hampshire, SO16 6YD, United Kingdom |
| Dr. Ashish Patwardhan | New Cross Hospital, Wolverhampton, West Midlands, WV10 0QP, United Kingdom |
| Dr. Benjamin Burton | James Paget University Hospital, Gorleston, Norfolk, NR31 6LA, United Kingdom |
| Mr. Bobby Paul | Queen's Hospital, Romford, Greater London, RM7 0AG, United Kingdom |
| Mr. Christopher Brand | Royal Hallamshire Hospital, Sheffield, South Yorkshire , S10 2JF, United Kingdom |
| Dr. Deepali Varma | Sunderland Eye Infirmary, Sunderland, Tyne & Wear, SR2 9HP, United Kingdom |
| Dr. Faruque Ghanchi | St Luke s Hospital Macula Clinic, Bradford, West Yorkshire, BD9 6RJ, United Kingdom |
| Ms. Geeta Menon | Frimley Park Hospital, Frimley, Surrey, GU16 7UJ, United Kingdom |
| Dr. Ian Pearce | Royal Liverpool University Hospital (Dept. St. Paul's Clinical Eye Research Centre), Liverpool, Norfolk, L7 8XP, United Kingdom |
| Mr. Javeed Khan | St Mary's Hospital, Newport, Isle of Wight, PO30 5TG, United Kingdom |
| Mr. Jignesh Patel | Essex County Hospital, Colchester, Essex, CO3 3NB, United Kingdom |
| Prof. Jonathan Gibson | Heart of England NHS Foundation Trust, Birmingham, West Midlands, B9 5SS, United Kingdom |
| Ms. Louise Downey | Hull and East Yorkshire Eye Hospital, Hull, North Yorkshire, HU32JZ, United Kingdom |
| Mr. Luke Membrey | Maidstone Hospital, Maidstone, Kent, ME16 9QQ, United Kingdom |
| Mr. Martin McKibbin | St James's University Hospital, Leeds, West Yorkshire, LS9 7TF, United Kingdom |
| Mr. Mohammed Musadiq | University Hospital of North Staffordshire Royal Infirmary, Stoke-on-Trent, Staffordshire, ST4 7LN, United Kingdom |
| Mr. Narendra Dhingra | Pinderfields Hospital GH, Wakefield, North Yorkshire, WF1 2DG, United Kingdom |
| Mr. Niral Karia | Southend University Hospital, Essex, SS0 0RY, United Kingdom |
| Mr. Nishal Patel | Kent and Canterbury Hospital, Kent, CT1 3NG, United Kingdom |
| Mr. Pankaj Puri | Royal Derby Hospital, Derby, Derbyshire, DE22 3NE, United Kingdom |
| Mr. Riaz Asaria | Royal Free Hospital, London, Greater London, NW3 2PF, United Kingdom |
| Mr. Richard Gale | York Hospital, York, North Yorkshire, YO31 8HE, United Kingdom |
| Mr. Rob Johnston | Cheltenham General Hospital, Cheltenham, Gloucestershire, GL54 7AN, United Kingdom |
| Mr. Robin Hamilton | Moorfields Eye Hospital, London, Greater London, EC1V 2PD, United Kingdom |
| Mr. Saad Younis | Western Eye Hospital, London, Greater London, NW1 5QH, United Kingdom |
| Mr. Sajjad Haider | University Hospital North Durham, Durham, DH1 5TW, United Kingdom |
| Mr. Sajjad Mahmood | Manchester Royal Eye Hospital, Manchester, Greater Manchester, M13 9WL, United Kingdom |
| Mr. Salman Mirza | Worcestershire Royal Hospital, Worcester, Worcestershire, WR5 1DD, United Kingdom |
| Mrs. Salwa Abugreen | Royal Blackburn Infirmary, Blackburn, Lancashire, BB2 3LR, United Kingdom |
| Mr. Samer El-Sherbiny | Birmingham and Midland Eye Centre, Birmingham, West Midlands, United Kingdom |
| Mr. Sanjiv Banerjee | University Hospital of Wales, Cardiff, CF14 4XW, United Kingdom |
| Mr. Sergio Pagliarini | Hospital St Cross Macular Unit, Rugby, West Midlands, CV225PH, United Kingdom |
| Ms. Sheena George | Hillingdon Hospital NHS Foundation Trust, Uxbridge, Greater London, UB8 3NN, United Kingdom |
| Dr. Simon Hewick | Raigmore Hospital, Inverness, Highland Region, IV2 3UJ, United Kingdom |
| Mr. Simon Kelly | Royal Bolton Hospital, Bolton, Lancashire, BL4 0JR, United Kingdom |
| Dr. Simon Taylor | Royal Surrey County Hospital, Guildford, Surrey, GU2 7XX, United Kingdom |
| Ms. Sohba Sivaprasad | King s College Hospital, London, Greater London, SE5 9RS, United Kingdom |
| Mr. Somnath Banerjee | Leicester Royal Infirmary, Leicester, Leicestershire, LE1 5WW, United Kingdom |
| Dr. Sudeshna Patra | Whipps Cross University Hospital, London, Greater London, E11 1NR, United Kingdom |
| Ms. Susan Downes | John Radcliffe Hospital, Oxford, Oxfordshire, OX3 7RP, United Kingdom |
| Dr. Tanya Moutray | Royal Victoria Hospital, Belfast, BT12 6 BA, United Kingdom |
| Mr. Tarek El-Kashab | Leighton Hospital, Crewe, Cheshire, CW1 4QJ, United Kingdom |
| Mr. Vineeth Kumar | Arrowe Park Hospital, Upton, Merseyside, CH49 5PE, United Kingdom |
| Mrs. Yinka Osoba | Torbay Hospital, Torquay, Devon, TQ2 7AA, United Kingdom |
| Dr. Yit Yang | New Cross Hospital, Wolverhampton, West Midlands, WV10 0QP, United Kingdom |
